# A “roller-wheel” Pt-containing small molecule that outperforms its polymer analogs in organic solar cells†
†Electronic supplementary information (ESI) available: Detailed synthetic procedures, DSC, XRD, absorption, CV and NMR spectra. CCDC 1478248. For ESI and crystallographic data in CIF or other electronic format see DOI: 10.1039/c6sc00513f


**DOI:** 10.1039/c6sc00513f

**Published:** 2016-05-23

**Authors:** Wenhan He, Maksim Y. Livshits, Diane A. Dickie, Jianzhong Yang, Rachel Quinnett, Jeffrey J. Rack, Qin Wu, Yang Qin

**Affiliations:** a Department of Chemistry & Chemical Biology , University of New Mexico , MSC03 2060, 1 UNM , Albuquerque , NM 87131 , USA . Email: yangqin@unm.edu; b Department of Chemical Engineering , Kansas State University , 1005 Durland Hall , Manhattan , KS 66506 , USA; c Center for Functional Nanomaterials , Brookhaven National Laboratory , PO Box 5000 , Upton , NY 11973 , USA

## Abstract

A novel Pt-bisacetylide small molecule (**Pt-SM**) featuring “roller-wheel” geometry was synthesized and characterized. When compared with conventional Pt-containing polymers and small molecules having “dumbbell” shaped structures, **Pt-SM** displays enhanced crystallinity and intermolecular π–π interactions, as well as favorable panchromatic absorption behaviors. Organic solar cells (OSCs) employing **Pt-SM** achieve power conversion efficiencies (PCEs) up to 5.9%, the highest reported so far for Pt-containing polymers and small molecules.

## Abstract 2

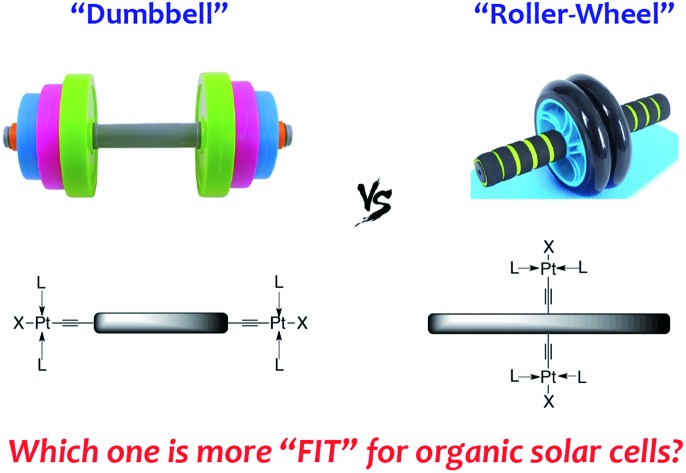
“Roller-wheel” shaped Pt-containing molecules display enhanced crystallinity and are better performing organic solar cell materials than conventional small molecules and polymers featuring “dumbbell” shaped structures.

## Introduction

**Scheme 1:**
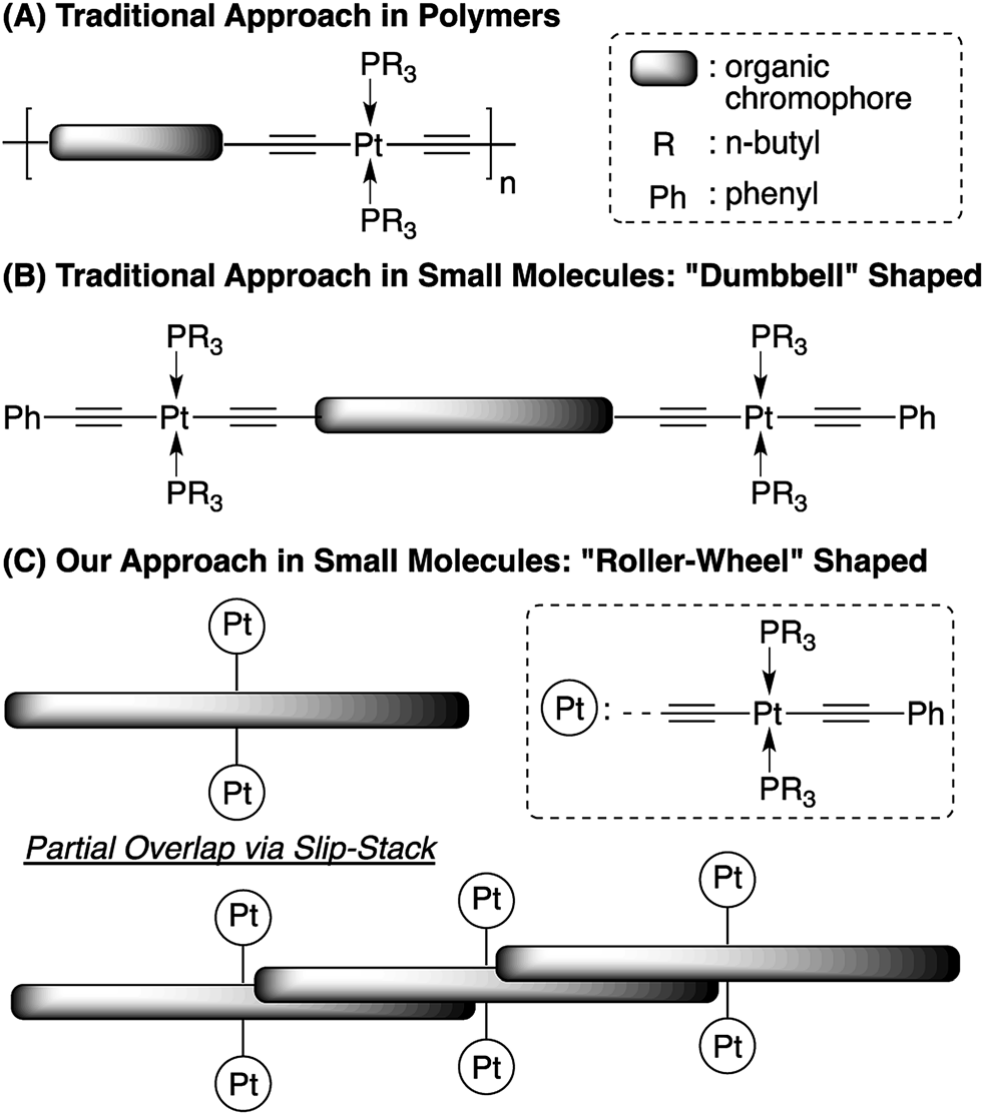
Structures of Pt-bisacetylide materials.

Organic solar cells (OSCs) have been widely perceived as low-cost alternative energy sources that possess unique properties including light-weight, flexibility and semi-transparency.^1^ State-of-the-art OSCs, with power conversion efficiencies (PCEs) now beyond 10%,^2^–^8^ contain blends of an organic light-absorbing electron donor and a fullerene derivative as the electron acceptor, forming bulk heterojunction (BHJ) morphologies that play a decisive role on device performance.^9^,^10^ Steady improvements in OSC efficiencies have resulted mainly from rational design and synthesis of conjugated donor materials,^11^,^12^ interfacial engineering,^13^,^14^ novel device geometries,^15^,^16^ as well as morphology optimization *via* Edisonian approaches including thermal/solvent annealing and additives.^17^,^18^ Another intriguing but less explored strategy is to incorporate heavy metals into the organic materials, leading to facile formation of triplet excitons. The extended lifetimes and thus longer diffusion lengths of triplet excitons have been suggested to improve the charge separation efficiency.^19^–^27^ Among the various examples of metal-containing materials applied in OSCs, conjugated polymers (CPs) containing main-chain Pt-bisacetylide moieties, having the general structures shown in Scheme 1A, are the most studied.^28^–^30^ Besides a handful of examples showing moderate PCEs of *ca.* 2–4%,^31^–^35^ most such Pt-containing CPs display relatively low performance, which has been mainly ascribed to their intrinsic amorphous nature, resulting in low conductivity and unfavorable BHJ morphologies.^36^–^39^

Although less explored in OSC applications, conjugated small molecules (SMs) can be highly crystalline and thus have superior charge mobilities, as well as discrete and reproducible molecular structures.^40^,^41^ These features have attracted increasing attention and OSC devices employing conjugated SMs have been constantly improved to rival their CP counterparts.^4^,^42^ On the other hand, conjugated SMs containing Pt-bisacetylides, having typical structures as shown in Scheme 1B, are mostly prepared as model compounds, resembling their polymeric analogs, for photophysical studies.^43^,^44^ These SMs, similar to their polymeric analogs, possess “dumbbell” shaped molecular structures having bulky Pt groups at both ends of linear organic chromophores, resulting in inefficient molecular packing and π–π interactions.

We propose a new structural design, as shown in Scheme 1C, having Pt-bisacetylides as side-chains attached at the center of a rigid and linear conjugated chromophore. This “roller-wheel” shaped structure can allow partial overlap among adjacent chromophores in a slip-stacked fashion similar to that observed in symmetrically substituted acenes,^45^,^46^ potentially enhancing crystallinity and conductivity. Herein, we report the synthesis, characterization and OSC application of such a “roller-wheel” molecule, **Pt-SM** that generates PCEs up to *ca.* 5.9% in BHJ devices, the highest reported so far for Pt-containing SMs and polymers.

## Results and discussion

**Scheme 2:**
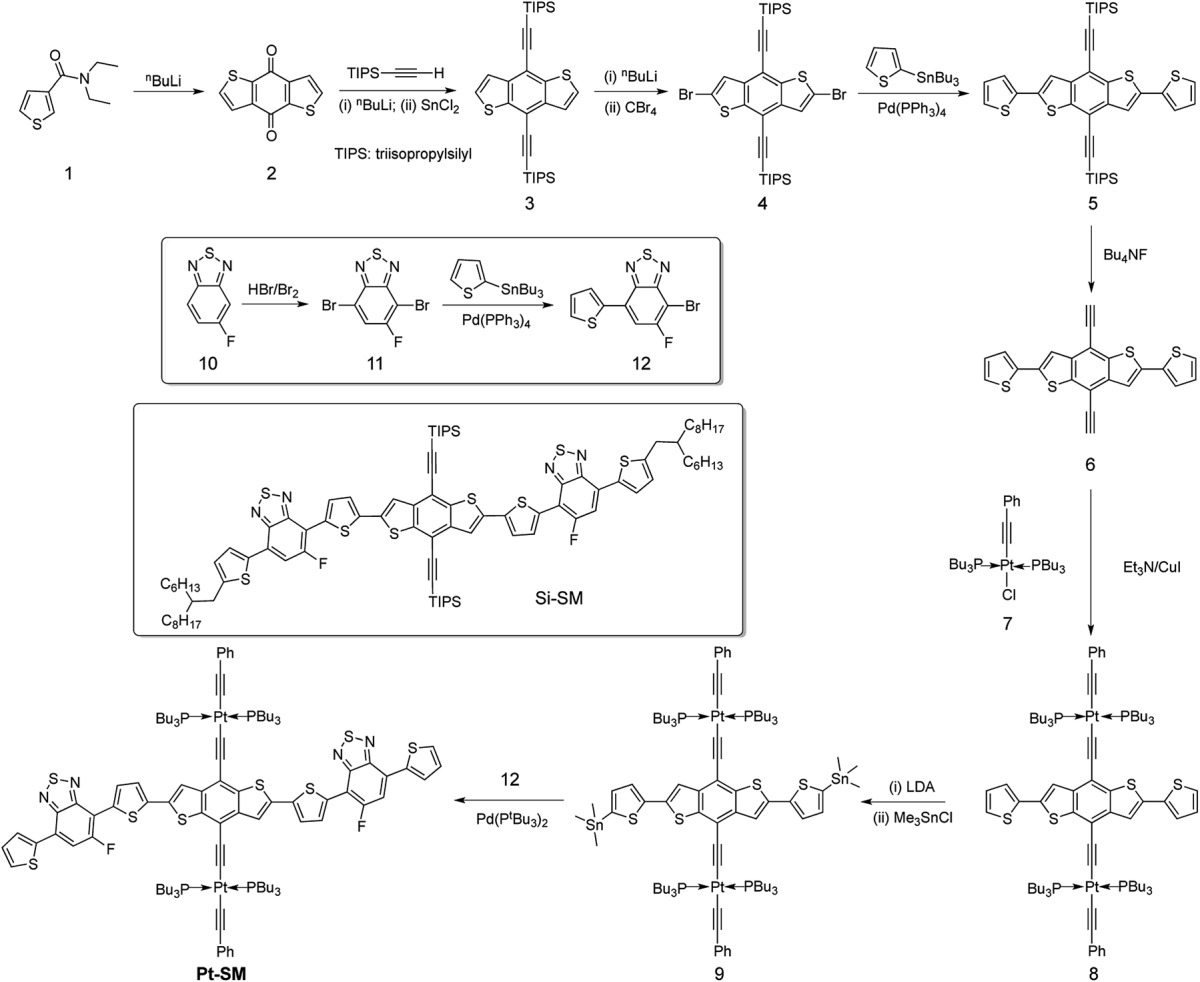
Synthesis of **Pt-SM** and structure of **Si-SM**.

Synthetic procedures toward the target compound **Pt-SM** are outlined in Scheme 2 and the structure of **Si-SM**, the non-metallic analog to **Pt-SM** by replacing the Pt-containing moieties with triisopropylsilyl (TIPS) groups is also shown. Synthetic details are included in the ESI.† The alkyl side-chains on **Si-SM** are necessary for solution processability, since attempts to prepare **Si-SM** analogs having no side-chains at both terminal thiophene rings led to materials that are poorly soluble in common organic solvents and difficult to purify and analyze. On the other hand, the four bulky tri(*n*-butyl)phosphine ligands make **Pt-SM** readily soluble in common organic solvents such as CHCl_3_, chlorobenzene, toluene and THF, *etc.*, without the need for additional alkyl side-chains. **Pt-SM** is fully characterized by ^1^H, ^13^C, ^19^F and ^31^P NMR spectroscopy, as well as high resolution mass spectrometry (HR-MS). Although we have not been able to obtain high quality single crystals for precise structure determination, certain crystallinity of **Pt-SM** can be observed in differential scanning calorimetry (DSC) and thin film X-ray diffraction (XRD) measurements. As shown in the DSC trace in Fig. S1A (see ESI†), a clear, reproducible melting transition peak at 114 °C is seen. Fig. S1B (see ESI†) displays several sharp XRD signals from **Pt-SM** films drop-cast onto glass substrates. Although accurate assignments of these peaks are difficult, the signal at 2*θ* = 23.3° corresponds to a *d*-spacing of *ca.* 3.81 Å, which is characteristic of typical π–π stacking distances of aromatic molecules.^47^ Such π–π stacking motifs are important for electronic coupling among adjacent chromophores and for efficient exciton/charge transport. Both the DSC and XRD measurements suggest that **Pt-SM** possesses certain crystallinity, which has rarely been observed in Pt-bisacetylide containing SMs and polymers.^48^

For a better comparison, we also studied the physical properties of the structurally similar **Si-SM** and compound **8** (Scheme 2) in detail. Fig. S2 (see ESI†) shows the DSC traces (2^nd^ heating) of these two compounds. Both **Si-SM** and **8** possess higher melting points at 243 °C and 204 °C, respectively, than that of **Pt-SM**. We have also not been able to obtain single crystals of **Si-SM** and its XRD pattern is shown in Fig. S3 (see ESI†). Only one scattering signal at 2*θ* = 4.18°, corresponding to a *d*-spacing of 2.11 nm, can be observed, which indicates less crystalline order for **Si-SM** likely caused by the large branched alkyl side-chains. On the other hand, we did obtain single crystals of compound **8** and the crystal structures and molecular packing motifs are displayed in Fig. S4 (see ESI†). Noticeably, although the conjugated chromophores are nearly planar and parallelly aligned, they are slip-stacked from each other along the short axis with tri-*n*-butylphosphine groups situated in between. Such packing geometry leads to an inter-chromophore distance of *ca.* 7.59 Å that is too large for efficient π–π interactions and charge transport (*vide infra*).

**Fig. 1:**
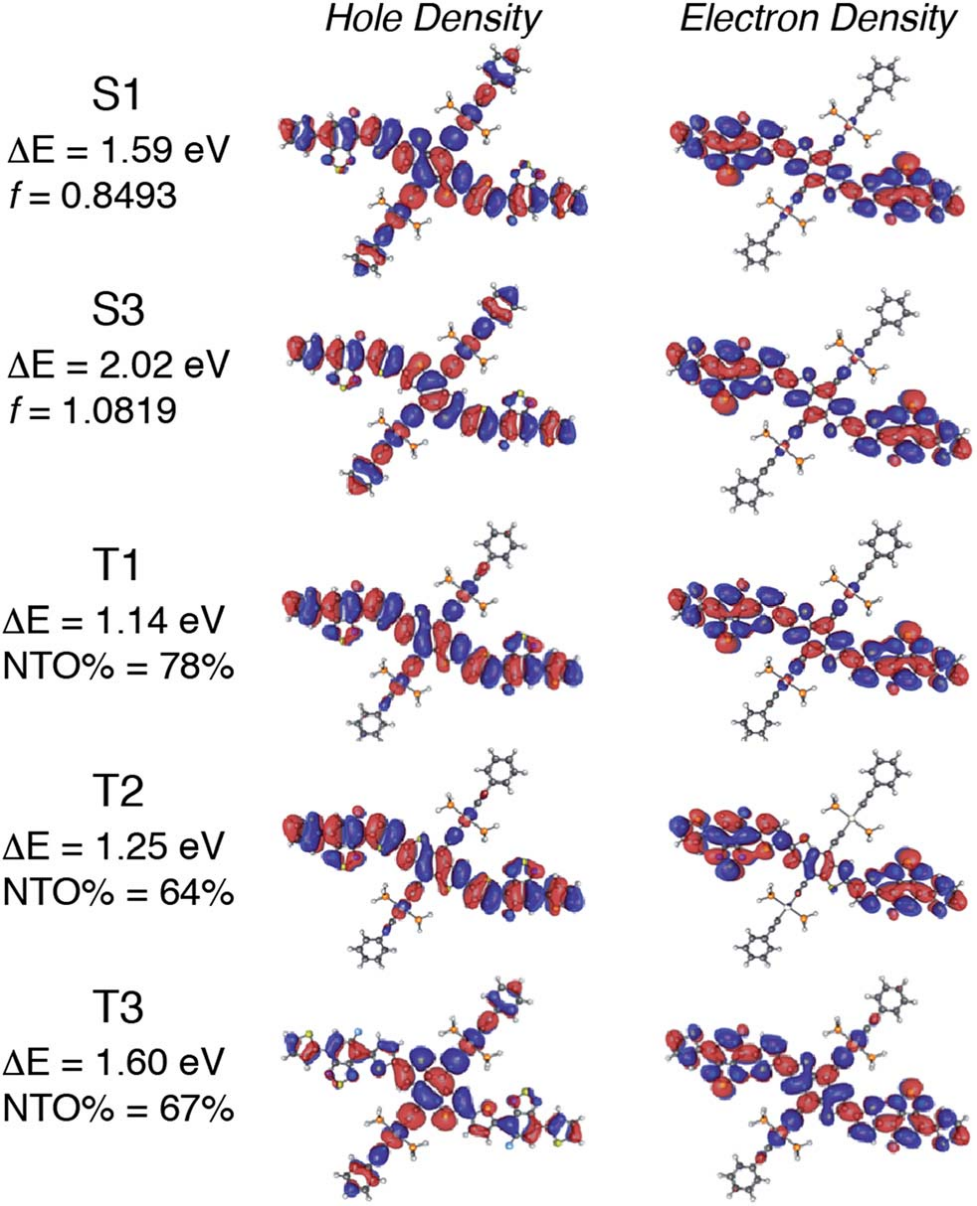
Low-lying bright singlet states and triplet states of **Pt-SM** calculated by density functional theory (DFT). Δ*E*: transition energy; *f*: oscillator strength; NTO: natural transition orbital.

The electronic properties of **Pt-SM** were first studied by using density functional theory (DFT) and the results are summarized in Fig. 1. All non-essential side chains are replaced with H atoms for computation efficiency and the ground state geometries are optimized using DFT, while the excited states are calculated with linear response time-dependent DFT (TD-DFT)^49^,^50^ at the optimized ground state geometries only. All calculations are performed with the Gaussian 09 package (Rev. B.01)^51^ using the hybrid B3LYP functional. The 6-31G* basis set is used for all atoms except for Pt, which has the LANL2DZ basis set for its 5s, 5p, 5d, and 6s valence electrons while the core electrons are replaced by the corresponding pseudopotential.^52^ A solvent reaction field simulated by the default polarizable continuum model (PCM) is also employed.^53^ We then use the natural transition orbital (NTO) approach^54^ to characterize the nature of the lowest singlet and triplet states. We first examined the bright singlet states in **Pt-SM** that are the S1 and S3 states having transition energies at 1.59 eV and 2.02 eV, respectively. From these NTOs that approximately represent the hole and electron in the transitions, both the S1 and S3 states have clear charge transfer characters mixed with metal-to-ligand-charge-transfer (MLCT) transitions, as expected from the electron rich benzenedithiophene (BDT) core and electron poor fluorinated benzothiadiazole (F-BTD) arms, as well as the hole density delocalized throughout the Pt-bisacetylide moieties. We then examine the triplet states in **Pt-SM**. It should be noted that the NTOs for these triplet states are only the dominant representation (given in percentages), unlike the singlet transition in Fig. 1 where the NTOs represent 100% of the transitions. There are two triplet states having energies lower than S1, which are the T1 (1.14 eV) and T2 (1.25 eV) states. The T1 state is delocalized along the organic chromophore while the T2 state has obvious charge transfer character with spin density more localized on the F-BTD moieties. The third triplet state, T3, lies at 1.60 eV, *i.e.*, less than 10 meV higher than that of S1. It is therefore plausible that an efficient intersystem crossing occurs at S1 and T3.^55^ T3 then fast decays into either T1 or T2, or both states, which then respectively decay to the ground state either single exponentially (from T1 or T2) or biexponentially (from both T1 and T2).

**Fig. 2:**
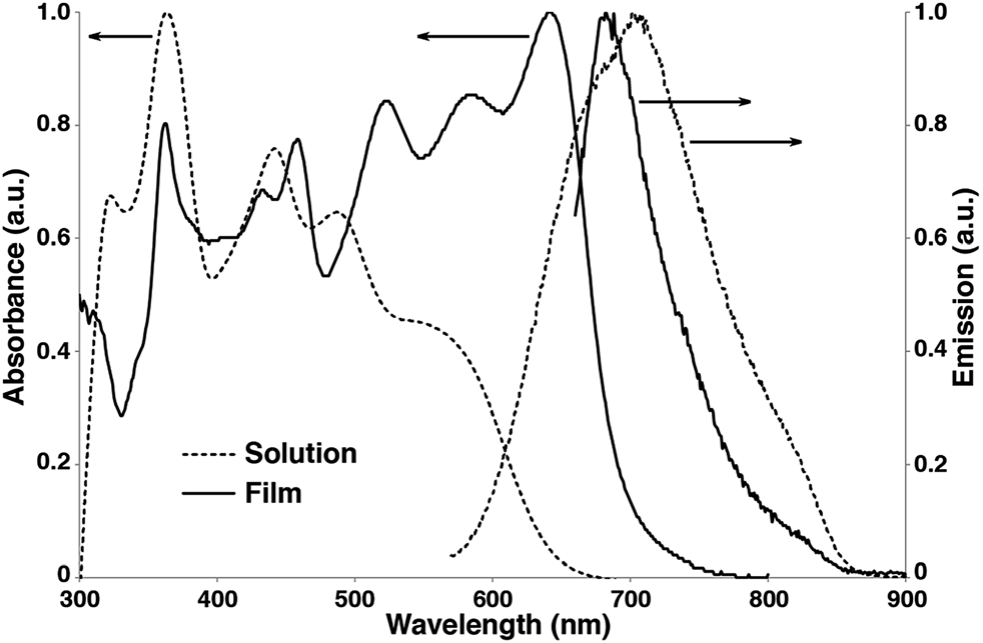
UV-vis absorption and emission spectra of **Pt-SM** in chlorobenzene solutions (10^–5^ M, dashed lines) and as thin films (solid lines).

The UV-vis absorption and emission spectra of **Pt-SM** in both dilute solutions and as thin films are shown in Fig. 2. Multiple absorption peaks at *ca.* 323, 364, 442, 487 and 560 nm are observed in dilute chlorobenzene solutions of **Pt-SM**. From the absorption onset at *ca.* 642 nm, the solution bandgap of **Pt-SM** is estimated to be *ca.* 1.93 eV. Upon casting into thin films, the absorption profile of **Pt-SM** experiences significant red-shifts, leading to an onset of *ca.* 692 nm and an estimated solid-state bandgap of *ca.* 1.79 eV. Intriguingly, the films of **Pt-SM** display panchromatic behavior with strong absorption covering a large spectroscopic range from 300 nm to 700 nm, having multiple peaks at *ca.* 362, 432, 458, 523, 583 and 642 nm. It is not clear at this stage whether these peaks may be vibronic in nature or if they originate from different electronic transitions as suggested by DFT calculations. This question merits more detailed studies in the future.

In order to qualitatively probe the origins of these electronic transitions, we also performed absorption and emission measurements of **Si-SM** and compound **8** under identical conditions. As shown in Fig. S5 (see ESI†), **Si-SM** displays a dominant structureless peak at 517 nm in solutions, becoming structured and red-shifted to 606 nm in thin films, which is absent for compound **8**. This low energy transition has been assigned to intramolecular charge-transfer (ICT) from the electron rich BDT centers to the electron poor F-BTD groups, as shown in recent examples with similar structures.^56^–^59^ We thus assign the low energy absorption peaks observed for **Pt-SM** to similar ICT transitions, which is consistent with our DFT calculations. Noticeably, these ICT transitions of **Pt-SM** are at lower energies respectively than those of **Si-SM**, suggesting electronic contribution to the ICT transitions from the Pt moieties through MLCT as suggested by the DFT calculations.

Furthermore, spectral red-shift from solution to thin film is only observed for **Pt-SM** and **Si-SM**, but not for **8**, indicating better molecular packing, and thus enhanced inter-molecular electronic interactions, for the first two compounds but otherwise for the latter. This observation is consistent with the XRD and crystal structure studies for **Pt-SM** and **8**, respectively. Compared with **8**, **Pt-SM** has identical bulky Pt-BDT center unit but more extended arms. The absorption profiles are thus consistent with our hypothesis that the “roller-wheel” structures can allow partial overlap and π–π interactions among adjacent molecules if the “roller-wheel” handles are long enough.

**Pt-SM** shows very weak emissions peaked at *ca.* 700 nm in solutions (excited at 560 nm) and at *ca.* 680 nm as thin films (excited at 642 nm), respectively. The slight blue shift of the emission in thin films from that of the solutions is presumably due to H-type aggregation of the chromophores,^60^,^61^ which is consistent with our conjecture on the solid-state structure of **Pt-SM**. The lifetime of these emissions was measured to be *ca.* 330 ps and did not change with or without the presence of oxygen. Also based on the relatively small Stokes shift, we assign the emissions of **Pt-SM** to fluorescence. The solution fluorescence quantum yield (QY) of **Pt-SM** is estimated to be *ca.* 0.77%, while the solution QY of **Si-SM** is calculated to be *ca.* 6.6%. This significant reduction in QY for **Pt-SM** is possibly caused by the attachment of Pt atoms, leading to increased intersystem crossing (ISC) rates. No phosphorescence could be observed for **Pt-SM** solutions or thin films even when cooled down to 10 K. Such lack of phosphorescence has been observed in previously reported Pt-containing low bandgap CPs and small molecules,^31^–^35^ which is commonly ascribed to the energy gap law.^62^ Noticeably, compound **8** does display a phosphorescence peak at *ca.* 705 nm in deaerated solutions that disappears when open to air (Fig. S5, see ESI†).

**Fig. 3:**
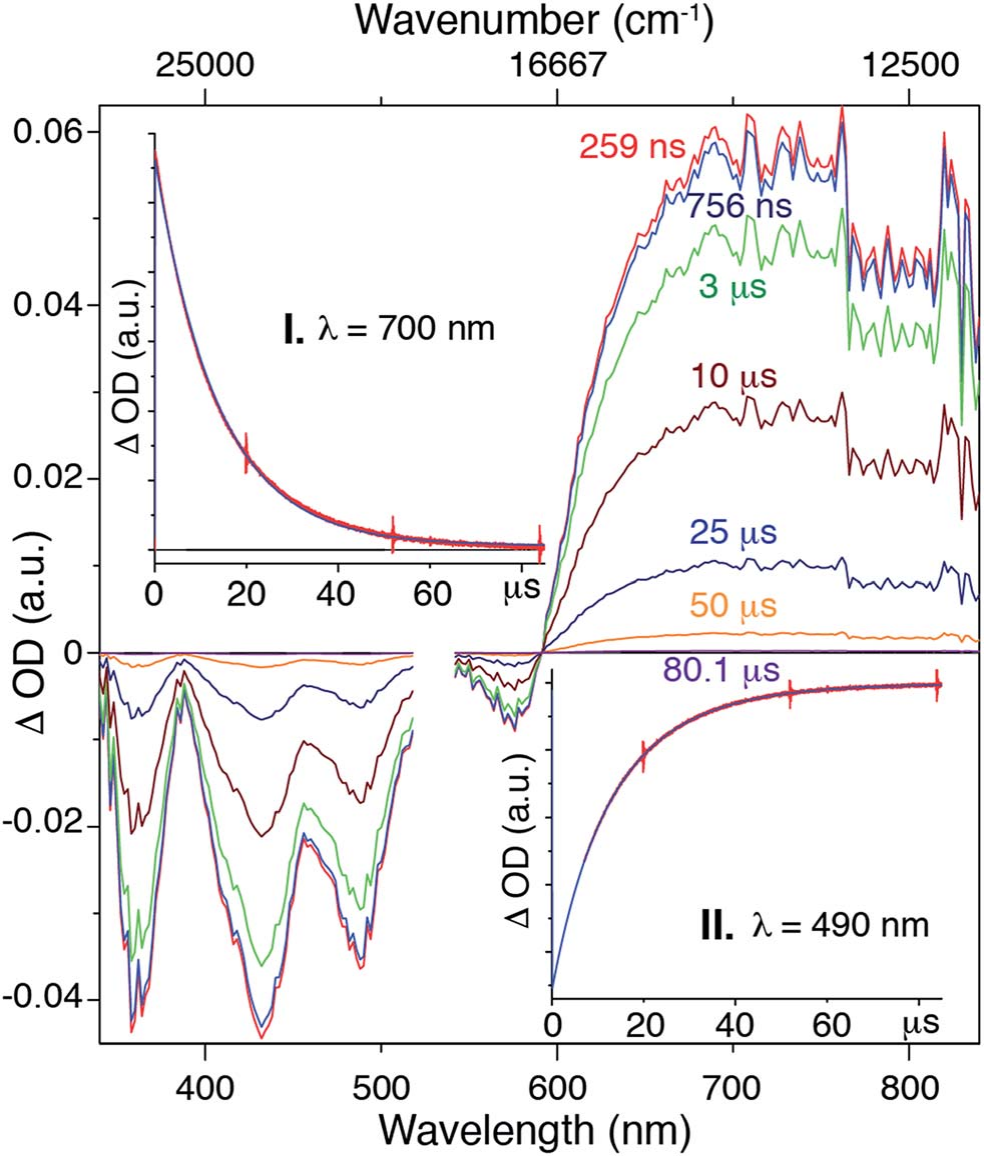
Transient absorption spectra of **Pt-SM** collected in chlorobenzene, excited at 532 nm with a nanosecond pulse from a SHG Continuum Surelight Nd : YAG at 1 Hz. The data near the excitation wavelength have been omitted for clarity. The transient spectrum was collected at 250 ns followed sequentially by transient spectra at 750 ns, 3 μs, 10 μs, 25 μs, 50 μs and 80 μs. Insert: single wavelength kinetic traces (red) and fits (blue) at 700 nm (I) and 490 nm (II).

Although we are not able to directly observe phosphorescence from **Pt-SM**, the presence of ISC events and triplet states have been demonstrated by using transient absorption spectroscopy as shown in Fig. 3. The 750 ns transient absorption spectrum of **Pt-SM** features four negative or bleach peaks from 340 nm to 600 nm, and a broad excited state absorption from 600 nm to 850 nm. The four bleach peaks are ascribed to loss of the ground state, as they are consistent with the absorption spectrum of **Pt-SM** (Fig. 2). We ascribe the excited state absorption feature to two different triplet transitions, as reported in similar systems,^24^,^25^,^30^,^37^ and supported by our DFT calculations. Single wavelength kinetics collected at 700 nm and 490 nm, as respectively shown in inserts I and II of Fig. 3, retrieved time constants of 7.5 ± 0.4 μs and 16.3 ± 0.4 μs. These are in good agreement with lifetimes of 7.6 ± 0.35 μs and 16.4 ± 0.27 μs, obtained from global fitting analysis. We ascribe the 7.5 μs time constant observed in the transient kinetics to a triplet charge separated state from the BDT core to the F-BTD arms, *i.e.*, the T2 state from DFT calculations. The 16.5 μs transition is thus ascribed to the T1 triplet state that is more delocalized. We make these assignments based upon independent transient absorption spectroscopic measurements of the compound **8** and **Si-SM**. The lifetime of the 700 nm transient absorption of **Si-SM** is fit to a single exponential of 1.6 μs and that of **8** (700 nm) is fit to a lifetime of 14.7 μs, respectively. This observation is consistent with DFT calculations, which find two triplet states of different origins in close energy (*ca.* 0.1 eV) to each other.

The HOMO and LUMO levels of **Pt-SM** were estimated using cyclic voltammetry (CV) as shown in Fig. S6 (see ESI†). One pseudo-reversible reduction and two oxidation peaks are observed, from the onsets of which, the HOMO and LUMO levels are estimated to be *ca.* –5.0 eV and –3.2 eV, respectively, leading to an electrochemical bandgap of *ca.* 1.8 eV that agrees well with the optical measurements.

One key parameter of organic materials for electronic devices is the charge mobility. We have measured hole mobilities of **Pt-SM**, **Si-SM** and compound **8** using space charge limited current (SCLC) method.^63^ Hole selective device geometries (ITO/MoO_3_/organic/MoO_3_/Al) are employed and the current density–voltage (*I*–*V*) curves are shown in Fig. S7 (see ESI†). From the linear region of each *I*–*V* curve, the hole mobilities of **Pt-SM**, **Si-SM** and **8** are estimated to be 1.5 × 10^–5^ cm^2^ V^–1^ s^–1^, 1.6 × 10^–5^ cm^2^ V^–1^ s^–1^ and 1.6 × 10^–6^ cm^2^ V^–1^ s^–1^, respectively. Although single crystalline in the solid state, **8** displays one order of magnitude lower hole mobility. This is expected from the crystal structure in which there is no efficient π–π overlap among adjacent chromophores for charge transport and agrees well with the nearly identical solution and film absorption profiles. On the other hand, **Pt-SM** and **Si-SM** show similarly enhanced charge mobilities, indicating better solid state packing structures and more efficient π–π stacking among adjacent conjugated chromophores.

OSC devices were fabricated using **Pt-SM**, **Si-SM** and compound **8** in device geometries: ITO glass/MoO_3_ (10 nm)/organic blend layer/Al (100 nm). The devices were first optimized using blends of phenyl-C_61_-butyric acid methyl ester (PC_61_BM) by varying the donor/acceptor weight ratios, solvent choices, solution concentrations, spin-coating conditions and thermal/solvent annealing conditions. Table S1† summarizes representative device parameters of **Pt-SM** from various optimization conditions and Table S2† gives the optimized device performances of **Si-SM** and **8** (see ESI†). The best devices were found to use a **Pt-SM**/PC_61_BM weight ratio of 10/8, the film thickness of *ca.* 80 nm and solvent annealing in CHCl_3_ saturated environment for 2 min. We then substituted PC_61_BM with phenyl-C_71_-butyric acid methyl ester (PC_71_BM) in the **Pt-SM** devices by employing the optimized conditions. Table 1 summarizes the performance parameters of the devices and Fig. 2 displays the representative *I*–*V* curves, and absorption and external quantum efficiency (EQE) profiles, as well as the transmission electron microscopy (TEM) image.

**Table 1:** Summary of OSC device performances employing **Pt-SM** and PC_71_BM^*a*^

Wt. ratio^*b*^	*J*_SC_ ^*c*^ (mA cm^–2^)	*V*_OC_ ^*d*^ (V)	FF^*e*^ (%)	PCE^*f*^ (%)	*R*_S_ ^*g*^ (Ω cm^2^)	*R*_SH_ ^*h*^ (Ω cm^2^)
10 : 8	11.9 ± 1.5 (14.4)	0.82 ± 0.03 (0.85)	57 ± 4.5 (63)	5.6 ± 0.29 (5.9)	5.6 ± 1.6	880 ± 520

^*a*^All devices employ **Pt-SM** and PC_71_BM, adopting device structures: ITO glass/MoO_3_ (10 nm)/organic blend layer (80 nm)/Al (100 nm), under simulated AM1.5 illumination. Average values and standard deviations are calculated from twelve individual devices, and the maximum values among tested devices are given in parentheses.

^*b*^Weight ratio of **Pt-SM**/PC_71_BM.

^*c*^Short circuit current.

^*d*^Open circuit voltage.

^*e*^Fill factor.

^*f*^Power conversion efficiency.

^*g*^Series resistance.

^*h*^Shunt resistance.

**Fig. 4:**
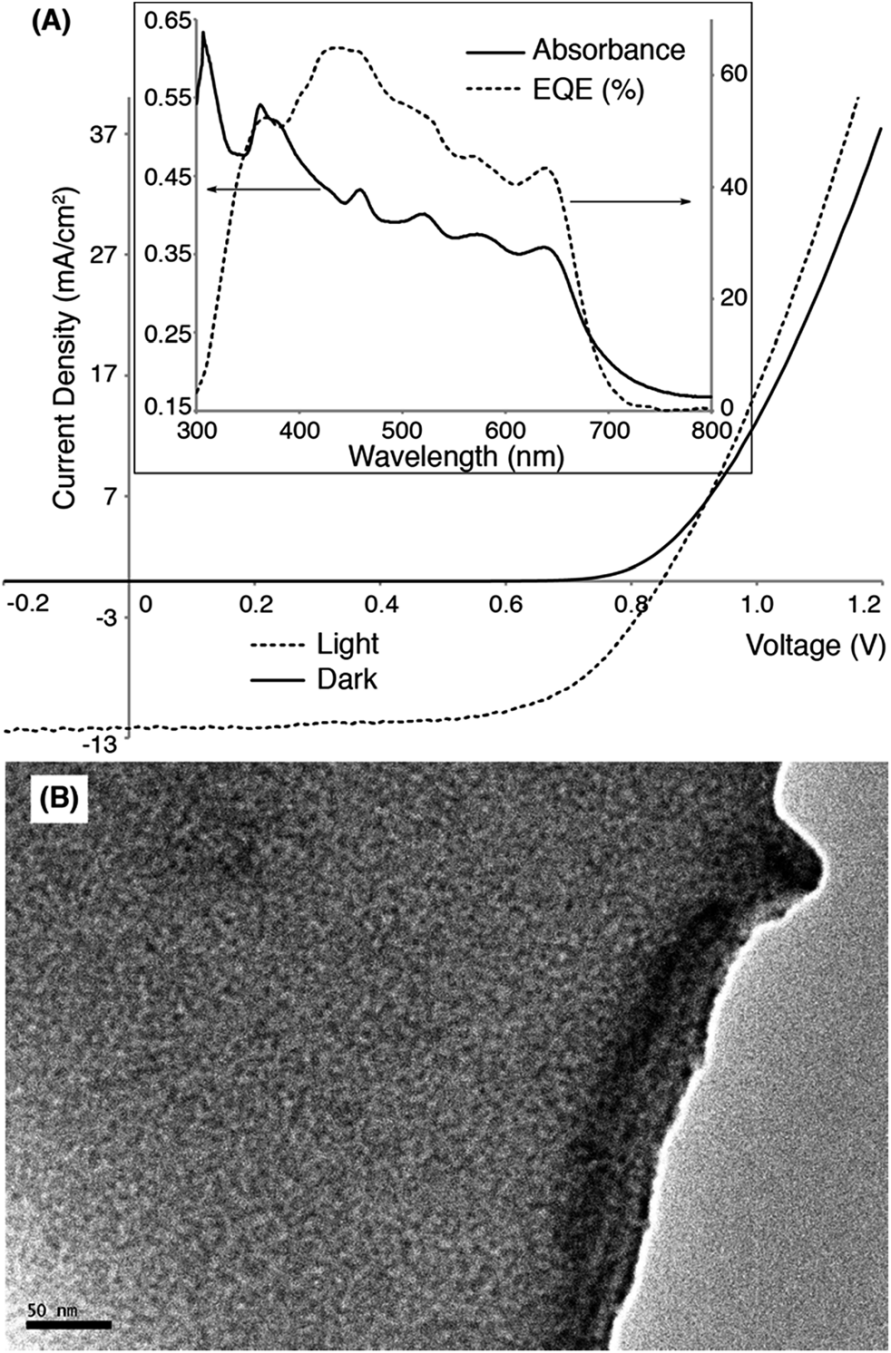
(A) Current density–voltage (*I*–*V*) curves of a representative OSC device in dark (solid line) and under simulated AM1.5 solar irradiation (dashed line). Insert: absorption (solid line) and external quantum efficiency (EQE, dashed line) profiles of the same device. (B) Transmission electron microscopy (TEM) image of the active layer of the device (scale bar is 50 nm).

The optimized devices display an average PCE of *ca.* 5.6% and a highest value of *ca.* 5.9%, which are significantly higher than values reported previously for OSCs applying Pt-containing polymers. The *J*_SC_ values of **Pt-SM** devices are comparable to those reported previously for best-performing Pt-containing polymers. The EQE responses closely match the thin film absorption profiles as shown in Fig. 4A (insert), displaying panchromatic responses from 350 nm to 650 nm. A relatively large *V*_OC_ value of *ca.* 0.82 V was obtained, indicating small energy loss during the charge separation and collection processes, considering that the energy gap between HOMO of **Pt-SM** and LUMO of PC_71_BM is *ca.* 1 eV.^64^ These *V*_OC_ values are also comparable with those from the best performing devices employing Pt-containing polymers.

The major improvement comes from the fill factors (FFs). **Pt-SM** devices constantly display FF*s* close to 60% while previous polymer examples rarely gave FF*s* above 40%. Furthermore, in previous examples, large excesses of fullerenes are required to reach the maximum PCEs due to the amorphous nature of the polymers. In the case of **Pt-SM**, only 44 wt% of fullerenes are sufficient to achieve the optimum performance, likely due to the more crystalline nature of the small molecule leading to better thin film morphologies. Indeed, TEM image of the device active layer (Fig. 4B) shows the absence of large aggregates and an interpenetrated network with favorable domain sizes of *ca.* 5–10 nm.

We then studied stability of the optimized **Pt-SM**/PC_71_BM devices through aging tests at room temperature and at 80 °C, and the results are summarized in Fig. S8 (see ESI†). After two weeks, the devices stored at room temperature show only slight decrease of performance to *ca.* 85% of the original PCEs; while devices kept at 80 °C display a bigger PCE drop to *ca.* 65%. From optical micrographs of these devices shown in Fig. S9 (see ESI†), both devices as optimized and aged at room temperature show smooth films free of any visible phase segregation and large aggregates. Phase separations on the order of a few μm can be observed in films aged at 80 °C after 14 days, which is still free of any large crystallites. Such coarsening of active layer morphologies likely explains the relatively higher decay rate of device performances.

## Conclusions

In summary, we have designed and synthesized a Pt-bisacetylide containing SM, featuring “roller-wheel” geometry. This new structural design allows partial overlap among the main rigid chromophores, leading to enhanced crystallinity and π–π interactions. OSCs employing **Pt-SM** displayed higher PCEs, mostly resulted from improved FFs, than those applying Pt-containing polymers reported previously. This molecular design strategy can be extended to other low bandgap linear organic chromophores as well as conjugated polymers, which will potentially lead to promising materials for OSCs and other organic electronics.

## Supplementary Material

Supplementary information

Crystal structure data
