# Supplementary material for: A “roller-wheel” Pt-containing small molecule that outperforms its polymer analogs in organic solar cells
Source: Chem Sci. 2016 May 23;7(9):5798–804. doi: 10.1039/c6sc00513f (PMC6021780; doi:10.1039/c6sc00513f)
Supplement: Supplementary file 1 [file SC-007-C6SC00513F-s001.pdf]

Electronic Supplementary Material (ESI) for Chemical Science.
This journal is © The Royal Society of Chemistry 2016

Electronic Supplementary Information

A "Roller Wheel" Pt-Containing Small Molecule that Outperforms Its Polymer Analogs in Organic Solar Cells

Wenhan He,[a] Maksim Y. Livshits,[a] Diane A. Dickie,[a] Jianzhong Yang,[a] Rachel Quinnett,[b] Jeffrey J. Rack,[a] Qin Wu[c] and Yang Qin*[a]

[a] Department of Chemistry & Chemical Biology, University of New Mexico, MSC03 2060, 1 UNM, Albuquerque, NM 87131, USA.

[b] Department of Chemical Engineering, Kansas State University, 1005 Durland Hall, Manhattan, KS 66506, USA.

[c] Center for Functional Nanomaterials, Brookhaven National Laboratory, PO Box 5000, Upton, NY 11973, USA.

** Corresponding author, e-mail: yangqin@unm.edu*

1. Materials and General Methods

All reagents and solvents were used as received from Sigma-Aldrich or VWR unless otherwise noted. Anhydrous THF was distilled over Na/benzophenone prior to use. Anhydrous chloroform and triethylamine (TEA) were obtained by distillation over CaH_2 and degassed through several freeze-pump-thaw cycles. Phenyl-C_{61}-butyric acid methyl ester ($PC_{61}BM$) and phenyl-C_{71}-butyric acid methyl ester ($PC_{71}BM$) were purchased from American Dye Source. N,N-Diethyl-3-thiophenecarboxamide (1),[1] benzo[1,2-b:4,5-b']dithiophene-4,8-dione (2),[2] benzo[1,2-b:4,5-b']dithiophene-4,8-bis[2-[triisopropylsilyl]ethynyl] (3),[3] 2,6-dibromo-4,8-bis[2-[triisopropylsilyl]ethynyl] (4),[4] benzo[1,2-b:4,5-b']dithiophene-2,6-di-2-thienyl-4,8-bis[2-[triisopropylsilyl]ethynyl] (5),[5] benzo[1,2-b:4,5-b']dithiophene-4,8-diethynyl-2,6-di-2-thienyl (6),[6] Trans-Chloro(phenylethynyl)bis(bributylphosphine)platinum(7),[7] 5-fluoro-2,1,3-benzothiadiazole (10),[8] 4,7-dibromo-5-fluoro-2,1,3-benzothiadiazole (11),[9] were synthesized according to literature procedures. 300.13 MHz ^{1}H, 75.48 MHz ^{13}C, 121.5 MHz ^{31}P and 282.4 MHz ^{19}F NMR spectra were recorded on a Bruker Avance III Solution 300 spectrometer. All solution ^{1}H spectra were referenced internally to tetramethylsilane and ^{13}C spectra were referenced internally to chloroform. ^{31}P and ^{19}F were referenced externally by using standards H_3PO_4 (δ = 0 ppm) and C_6F_6 (δ = −164.9 ppm). Ultraviolet visible (UV-vis) absorption spectra were recorded on a Shimadzu UV-2401 PC spectrometer over a wavelength range of 300−900 nm. Fluorescence emission spectra were obtained using a Varian Cary Eclipse Fluorimeter. Fluorescence quantum yield (QY) is estimated using quinine sulfate as a standard (54% in 0.1 M H_2SO_4).[10] Differential scanning calorimetry (DSC) measurements were performed on a Mettler Toledo DSC STARe system with ca. 8 mg sample and at a scan rate of 10 °C/min. The results reported are from the second heating cycle. Cyclic voltammetry (CV) was performed at 25 °C on a CH Instrument CH1604xD electrochemical analyzer using a glassy carbon working electrode,

a platinum wire counter electrode and Ag/AgCl reference electrode, calibrated using a ferrocene redox couple (4.8 eV below vacuum). X-ray diffraction (XRD) data was collected using a Rigaku SmartLab diffractometer in Bragg-Brentano mode employing Cu K-α radiation and a D/tex 1-dimensional detector. A nickel filter was used to remove the Cu K-β radiation component. Data was collected over a 2θ range from 5° to 40° using 0.02° step size at a scan rate of 1°/min. Transmission electron microscopy (TEM) images were taken on a JEOL 2010 EX HREM with an Oxford-Link EDS Gatan Digital Micrograph equipped with a slow scan CCD camera. Samples were prepared by soaking solar cell devices employing PEDOT:PSS as anode interfacial layers in water and fishing our floating active layers with carbon coated TEM grids. High resolution mass spectrometry (HRMS) was analyzed by Mass Spectrometry using Electrospray Ionization (ESI) in positive mode on a Waters LCT Premier Time of Flight (TOF) mass spectrometer. Thin film thickness was measured using the KLA-Tencor D-100 Profilometer. Transient absorption lifetimes were collected on an Edinburgh Instruments LP920 Laser Flash Photolysis Spectrometer. The excitation source consists of a Continuum Surelight II Model SLI-10 (Nd:YAG, 10 Hz) with SHG and THG options to generate 532 nm and 355 nm wavelengths. A Surelight SSP (dichroic to separate the 532 and 355) is positioned in between the laser to select the desired pump wavelength. A 450W ozone free Xenon arc lamp generates a microsecond white light supercontinuum probe beam, which is arranged in a 90° cross-beam geometry excitation beam at the sample. The probe beam passes through a monochromator and is captured by a Hamamatsu R928 PMT. Individual single wavelength kinetics are collected at a 2 nm step size to construct transient absorption spectra at specific times. Transient data are fit by re-convolution in SurfaceXplorer (v. 4) using a second order multiexponential decay with a fixed Instrument Response Function of 40 ns.

2. Solar Cell Fabrication and Testing

Pt-SM/$PC_{71}BM$ blend solutions were prepared by dissolving **Pt-SM** and $PC_{71}BM$ at predetermined weight ratios in chloroform at a **Pt-SM** concentration of 0.5 wt% and stirred at room temperature for 3-4 hours in a nitrogen glovebox (Innovative Technology, model PL-He-2GB, O_2 < 0.5 ppm, H_2O < 0.5 ppm). Solar cell devices were fabricated according to the following procedure: ITO-coated glass substrates (China Shenzhen Southern Glass Display Ltd.; 8 Ω/□) were cleaned by ultrasonication sequentially in detergent water, DI water, acetone and isopropyl alcohol, each for 15 min. These ITO-coated glasses were further treated by UV-ozone (PSD Series, Novascan) for 60 min before being transferred to a nitrogen glovebox (Innovative Technology, model PL-He-2GB, O_2 < 0.1 ppm, H_2O < 0.1 ppm) for MoO_3 deposition. MoO_3 (10 nm) was deposited using an Angstrom Engineering Amod deposition system at a base vacuum level of $< 4\times10^{-7}$ Torr. The **Pt-SM**/fullerene blend solution was first filtered through a 0.45 μm PTFE filter and spin-coated on top of the MoO_3 layer at 1200 rpm for 30 s. Al (100 nm) was thermally evaporated through patterned shadow masks as anodes, the sizes of which define the active areas of the solar cells to be 7.1 mm^2. Current-voltage (I-V) characteristics were measured using a Keithley 2400 source-measuring unit under simulated AM 1.5G irradiation (100 mW/cm^2) generated by a Xe acr-lamp based Newport 67005 150 W solar simulator equipped with an AM 1.5G filter. The light intensity was calibrated using a Newport thermopile detector (model 818P-010-12) equipped with a Newport 1916-C optical Power Meter. External quantum efficiency (EQE) values were measured by using a commercial solar cell quantum efficiency measurement system (Model QEXL, PV Measurements, Inc., Boulder, CO). The EQE system

was calibrated with a Si photodiode certified by the National Renewable Energy Laboratory (NREL).

3. Synthetic Scheme

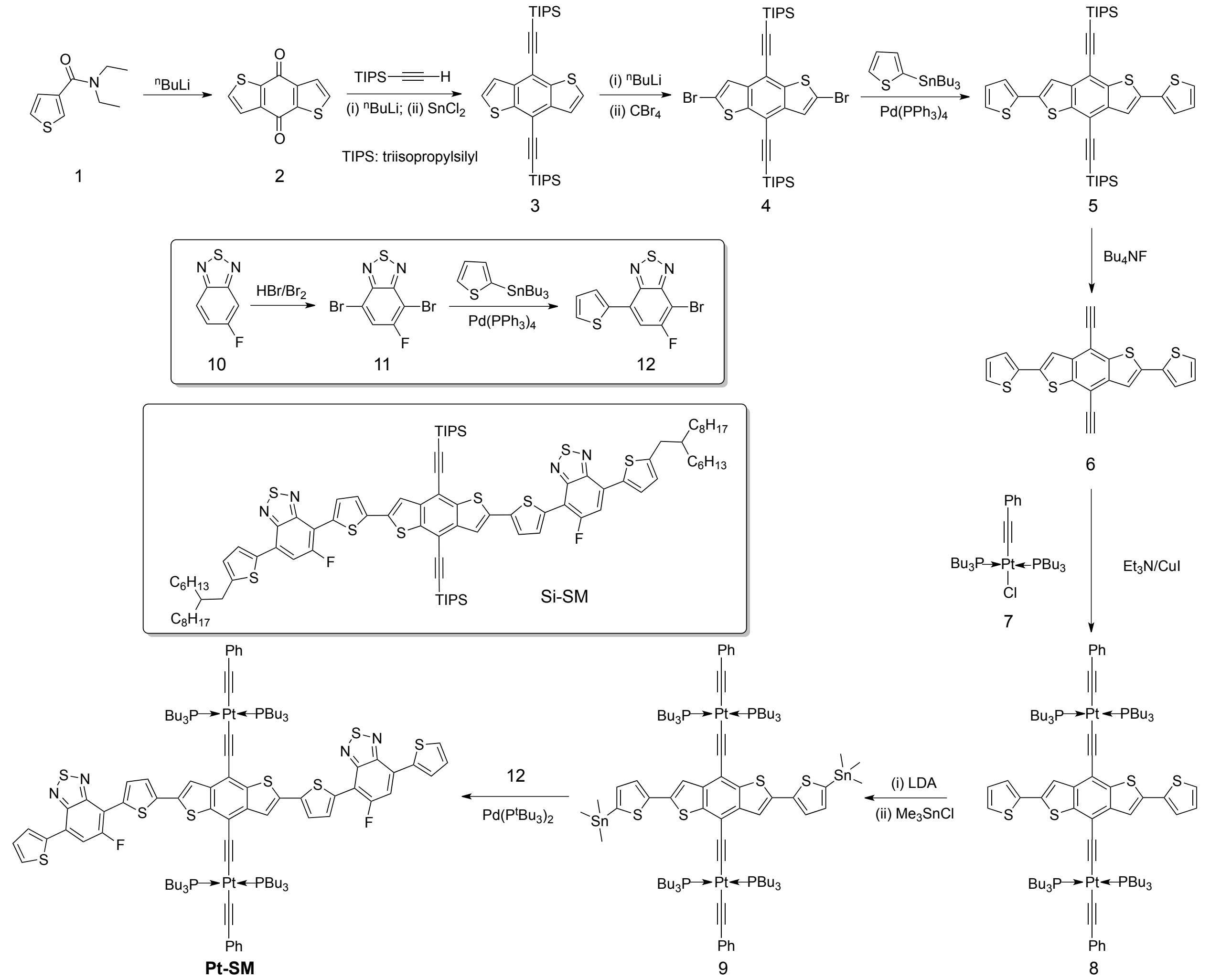

4. Experimental Details

Compound 8. Into a 100 mL round bottom flask equipped with a magnetic stir bar, compound 6 (185 mg, 0.46 mmol), compound 7 (812 mg, 1.1 mmol) and CuI (17.5 mg, 0.092 mmol) were dissolved in a mixture of 40 mL anhydrous chloroform and 2 mL triethylamine under argon. The flask was then sealed and the reaction mixture was stirred for 12 h before removal of the solvents under reduced pressure. The product **8** was isolated by flash column chromatography using hexanes/CH_2Cl_2 (8/1) and further purified by recrystallization from $CHCl_3$/CH_3OH mixture as a yellow solid (700 mg, 85% yield). ^{1}H NMR (300.13 MHz, $CDCl_3$): δ (ppm) = 7.66 (s, 2H), 7.32 (m, 6H), 7.21 (m, 6H), 7.12(m, 2H), 7.04 (dd, 2H, $^3J_{HH}$ = 3.6 Hz), 2.21 (m, 24H), 1.66 (m, 24H), 1.44 (m, 24H), 0.91 (t, 36H, $^3J_{HH}$ = 7.2 Hz). ^{31}P NMR (121.5 MHz, $CDCl_3$): δ (ppm) = 4.03 ($J_{Pt\text{-}P}$ = 2,350 Hz). ^{13}C NMR (75.48 MHz, $CDCl_3$): δ (ppm) = 138.7, 138.2, 136.0, 130.9, 129.1, 127.8, 127.7, 124.8, 124.1, 120.4, 120.1, 114.2, 109.6, 107.8,

105.3, 26.5, 24.3, 23.9, 13.9. Orange rod like single crystals of $C_{86}H_{126}P_4Pt_2S_4$, **8**, approximate dimensions 0.130 mm x 0.144 mm x 0.192 mm, was coated with Paratone oil and mounted on a MiTeGen MicroLoop that had been previously attached to a metallic pin using epoxy for the X-ray crystallographic analysis. The X-ray intensity data were measured on a Bruker Kappa APEX II CCD system equipped with a graphite monochromator and a Mo Kα fine-focus tube (λ = 0.71073 Å). Crystal data. $C_{86}H_{126}P_4Pt_2S_4$, M = 1802.16, monoclinic, a = 12.7253(4), b = 8.8911(3), c = 37.7183(12) Å, β = 94.230(2)°, U = 4255.9(2) Å^3, T = 99(2) K, space group $P\ 21/n$, Z = 2, 42733 reflections measured, 10187 unique (Rint = 0.0871), which were used in all calculations. The final w$R(F2)$ was 0.0986 (all data).

Compound 9. Into a solution of compound **8** (480 mg, 0.27 mmol) in dry THF (20 mL) being stirred under nitrogen at −78 °C, was added slowly 0.4 mL lithium diisopropylamide (2.0 M in THF/n-heptane/ethylbenzene, 0.8 mmol). After stirring for 60 min at −78 °C, 186 mg trimethyltin chloride (0.93 mmol) dissolved in 5 mL dry THF was added dropwise and the reaction mixture was slowly warmed up to room temperature and further stirred for 12 h. The reaction was quenched by the addition of water and extracted with chloroform (×3). The organic layer was washed with water, brine and dried over anhydrous Na_2SO_4. After the solvent was removed under vacuum, the crude product was purified by recrystallization ($CHCl_3$/MeOH) as a yellow solid (500 mg, 88% yield). ^{1}H NMR (300.13 MHz, $CDCl_3$): δ (ppm) = 7.66 (s, 2H), 7.32 (m, 6H), 7.21 (m, 4H), 7.11(m, 4H), 2.20 (m, 24H), 1.67 (m, 24H), 1.45 (m, 24H), 0.91 (t, 36H, $^3J_{HH}$ = 7.2 Hz), 0.39 (s, 18H). ^{31}P NMR (121.5 MHz, $CDCl_3$): δ (ppm) = 4.01 ($J_{Pt\text{-}P}$ = 2,352 Hz) ^{13}C NMR (75.48 MHz, $CDCl_3$): δ (ppm) = 144.3, 138.7, 138.2, 137.9, 135.9, 135.8, 130.8, 129.1, 127.8, 125.3, 124.8, 120.0, 114.0, 109.5, 107.9, 105.3, 26.5, 24.4, 23.9, 13.9, −8.30.

Compound 12. Into a 125 mL pressure vessel, were added compound 11 (1.0 g, 3.2 mmol), tributyl(thiophen-2-yl)stannane (1.2 g, 3.2 mmol), tetrakis(triphenylphospine)palladium (111 mg, 0.1 mmol) and 30 mL dry toluene, and the vessel was sealed and stirred at 120 °C for 24 hours. Toluene was then removed under reduced pressure and the residue was dissolved in chloroform. The solution was passed through a short silica gel column and volatile solvents were removed under reduced pressure. The crude product was purified by flash column chromatography using hexanes/CH_2Cl_2 (10/1) and the product 12 was further purified by recrystallization ($CHCl_3$/MeOH) as an orange red solid (706 mg, 70% yield). ^{1}H NMR (300.13 MHz, $CDCl_3$): δ (ppm) = 8.13 (d, 1H, $^3J_{HH}$ = 3.6 Hz), 7.74 (d, 1H, $^3J_{HH}$ = 10.2 Hz), 7.54 (d, 1H, $^3J_{HH}$ = 5.1 Hz), 7.22 (dd, 1H, $^3J_{HH}$ = 3.9 Hz). ^{19}F NMR (282.4 MHz, $CDCl_3$): δ (ppm) = −101.8. ^{13}C NMR (75.48 MHz, $CDCl_3$): δ (ppm) = 162.2, 158.9, 154.0, 148.8, 137.1, 128.8, 128.3, 127.4, 116.3, 96.2.

Pt-SM. Compound 9 (200 mg, 0.094 mmol) and compound 12 (177 mg, 0.564 mmol) were dissolved in 20 mL anhydrous toluene in a 100 mL flask under argon, and Pd(^{t}Bu$_3$P)$_2$ (9.6 mg, 0.019 mmol) was added as a solid. The flask was sealed and the reaction mixture was stirred for six hours at room temperature. Toluene was then removed under reduced pressure and the resulting crude product was purified by column chromatograph using hexanes/CH_2Cl_2 (4/1). **Pt-SM** was further purified by recrystallization ($CHCl_3$/MeOH) as a dark red solid (35 mg, 16.4% yield). ^{1}H NMR (300.13 MHz, $CDCl_3$): δ (ppm) = 8.31 (d, 2H, $^3J_{HH}$ = 3.9 Hz), 8.17 (dd, 2H, $^3J_{HH}$

= 3.6 Hz), 7.83 (s, 2H), 7.80 (d, 2H, $^3J_{HH}$ = 12.9 Hz), 7.53 (dd, 2H, $^3J_{HH}$ = 4.8 Hz), 7.36 (m, 6H), 7.15 (m, 8H), 2.24 (m, 24H), 1.71 (m, 24H), 1.49 (m, 24H), 0.94 (t, 36H, $^3J_{HH}$ = 7.2 Hz). ^{31}P NMR (121.5MHz, $CDCl_3$): δ (ppm) = 4.05 ($J_{Pt\text{-}P}$ = 2,345 Hz). ^{19}F NMR (282.4 MHz, $CDCl_3$): δ (ppm) = −106.2. ^{13}C NMR (75.48 MHz, $CDCl_3$): δ (ppm) = 160.7, 157.3, 153.4, 149.9, 141.1, 139.2, 138.5, 138.0, 135.9, 132.1, 131.2, 131.1, 130.9, 128.4, 127.9, 124.8, 124.5, 120.7, 117.4, 114.5, 111.2, 109.6, 107.8,105.4, 26.5, 24.4, 24.0, 13.9. HRMS: (ESI): $C_{106}H_{132}F_2N_4P_4Pt_2S_8$, calcd, 2269.6510 for [$M^+$]; found, 2269.6453 for [M^+].

5. References

1. Schröder, N.; Lied, F.; Glorius, F. *J. Am. Chem. Soc.* **2015,** *137,* 1448.
2. Pawle, R. H.; Agarwal, A.; Malveira, S.; Smith, Z. C.; Thomas, S. W. *Macromolecules* **2014,** *47,* 2250.
3. Kim, H. G.; Jo, S. B.; Shim, C.; Lee, J.; Shin, J.; Cho, E. C.; Ihn, S. G.; Choi, Y. S.; Kim, Y.; Cho, K. *J. Mater. Chem.* **2012,** *22,* 17709.
4. Lin, Y. Z.; Yeh, C. W.; Chou, P. T.; Watanabe, M.; Chang, Y. H.; Chang, Y. J.; Chow, T. J. *Dyes and Pigments* **2014,** *109,* 81.
5. Taybet, B.; Kerstin, S.; Tatjana, E. B.; Katrin, F.; Silvia, J. *Organic Electronics* **2013,** *14,* 344.
6. Wu, Y. L.; Liu, P.; Wigglesworth, A. J.; Hu, N. X. U.S. Pat. Appl. Publ. 2011, US 20110260114 A1 20111027
7 Dubinina, G. G.; Price, S. R.; Abboud, A. K.; Wicks, G.; Wnuk, P.; Stepanenko, Y.; Drobizhev, M.; Rebane, A.; Schanze, S. K. *J. Am. Chem. Soc.* **2012,** *134,* 19346.
8. Stuart, A. C.; Tumbleston, J. R.; Zhou, H. X.; Li, W. T.; Liu, S. B.; Ade, H.; You, W. *J. Am. Chem. Soc.* **2013,** *135,* 1806.
9. Kim, J. G.; Yun, M. H.; Kim, G. H.; Lee, J.; Lee, S. M.; Ko, S. J.; Kim, Y.; Dutta, G. K.; Moon, M.; Park, S. Y.; Kim, D. S.; Kim, J. Y.; Yang, C. *ACS Appl. Mater. Interfaces* **2014,** *6,* 7523.
10. Melhuish, W. H. *J. Phys. Chem.* **1961**, *65*, 229.

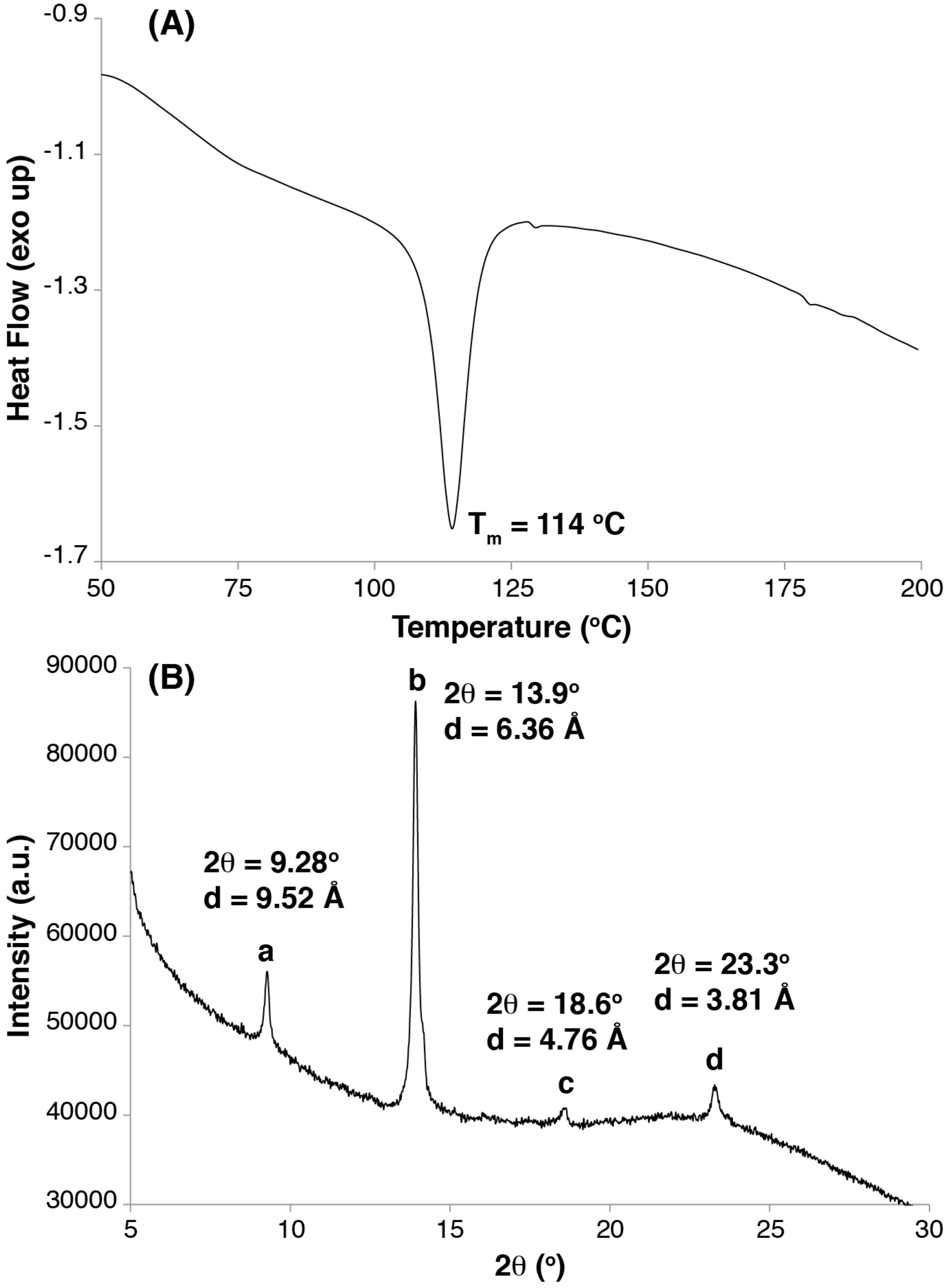

Figure S1. (A) Differential scanning calorimetry (DSC) trace (2nd heating, 10 °C/min) of **Pt-SM**. (B) X-ray diffraction (XRD) patterns of **Pt-SM** films drop-cast onto glass substrates.

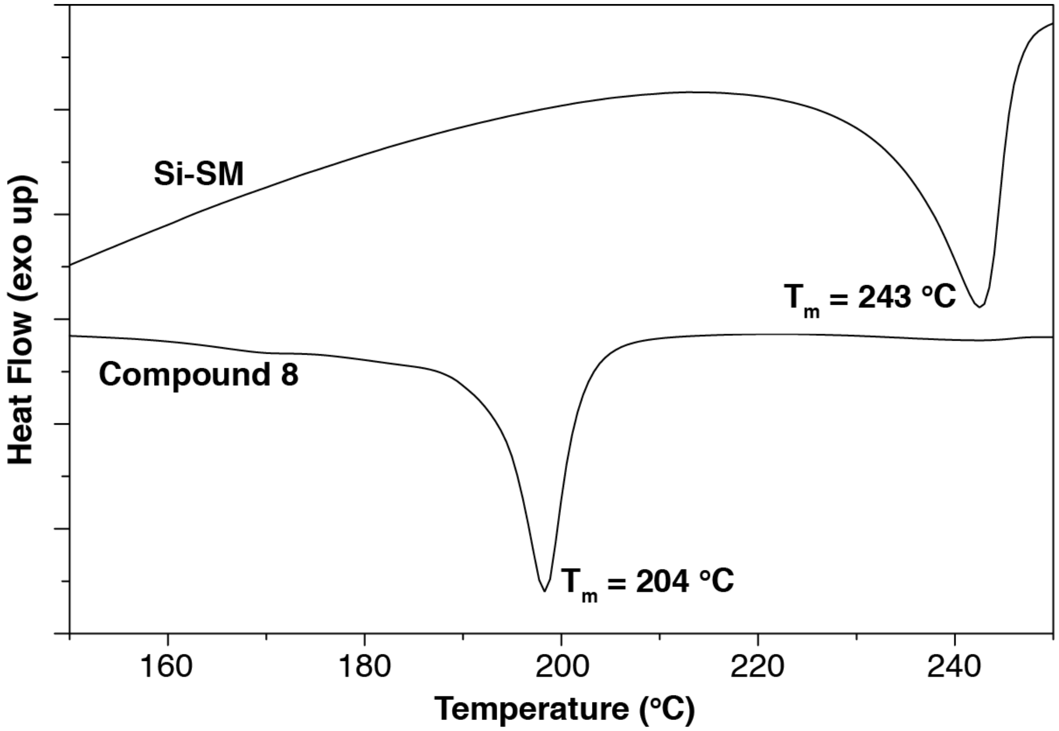

Figure S2. Differential scanning calorimetry (DSC) trace (2nd heating, 10 °C/min) of **Si-SM** and compound **8**.

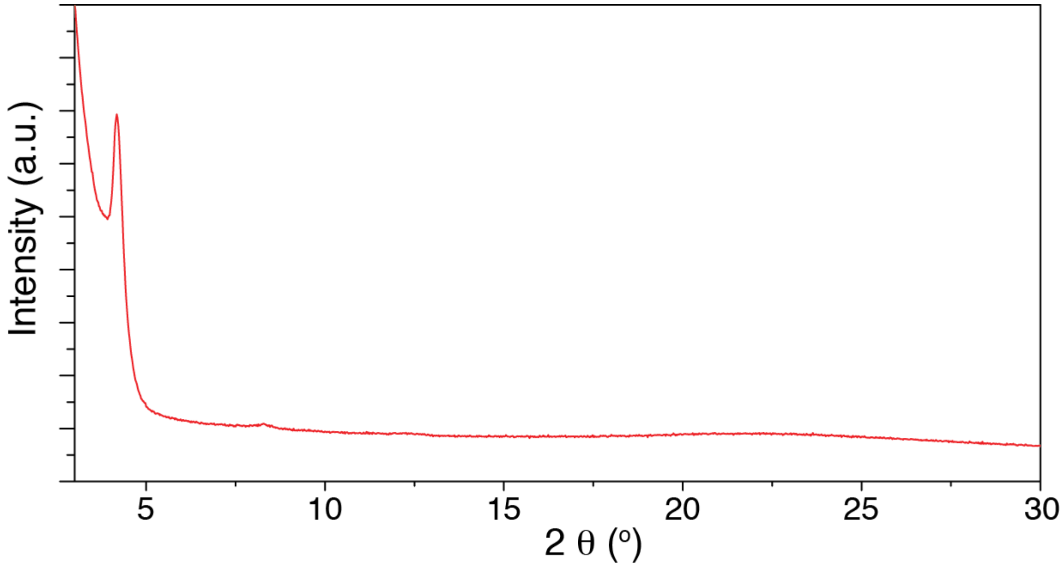

Figure S3. X-ray diffraction (XRD) patterns of **Si-SM** films drop-cast onto glass substrates.

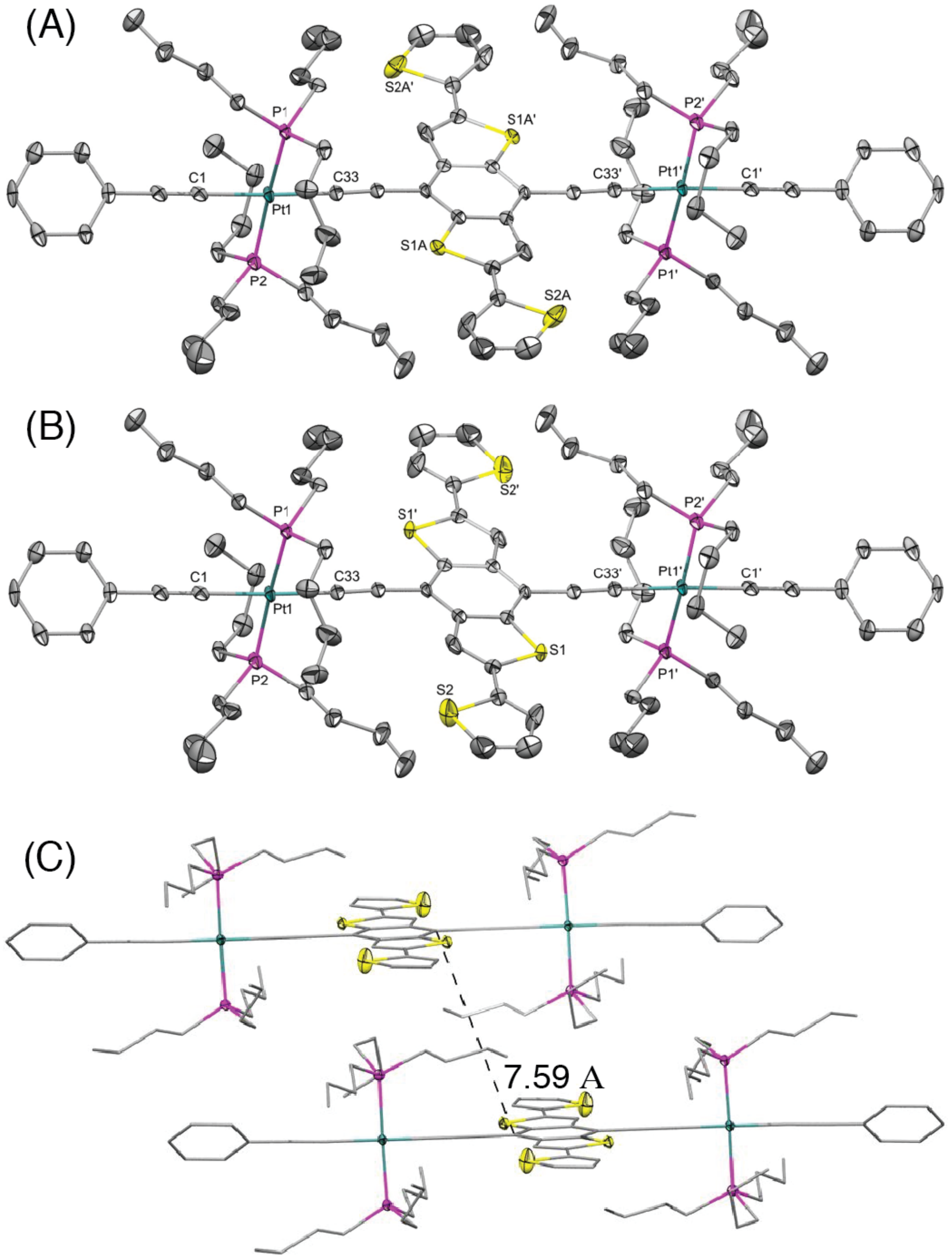

Figure S4. Crystal structures of **8**. Thermal ellipsoids are drawn at 50% probability and hydrogen atoms have been omitted for clarity. (A) Major configuration (ca. 73%). (B) Minor configuration (ca. 27%). (C) Packing diagram of the major configuration with distance between C35-C35 shown.

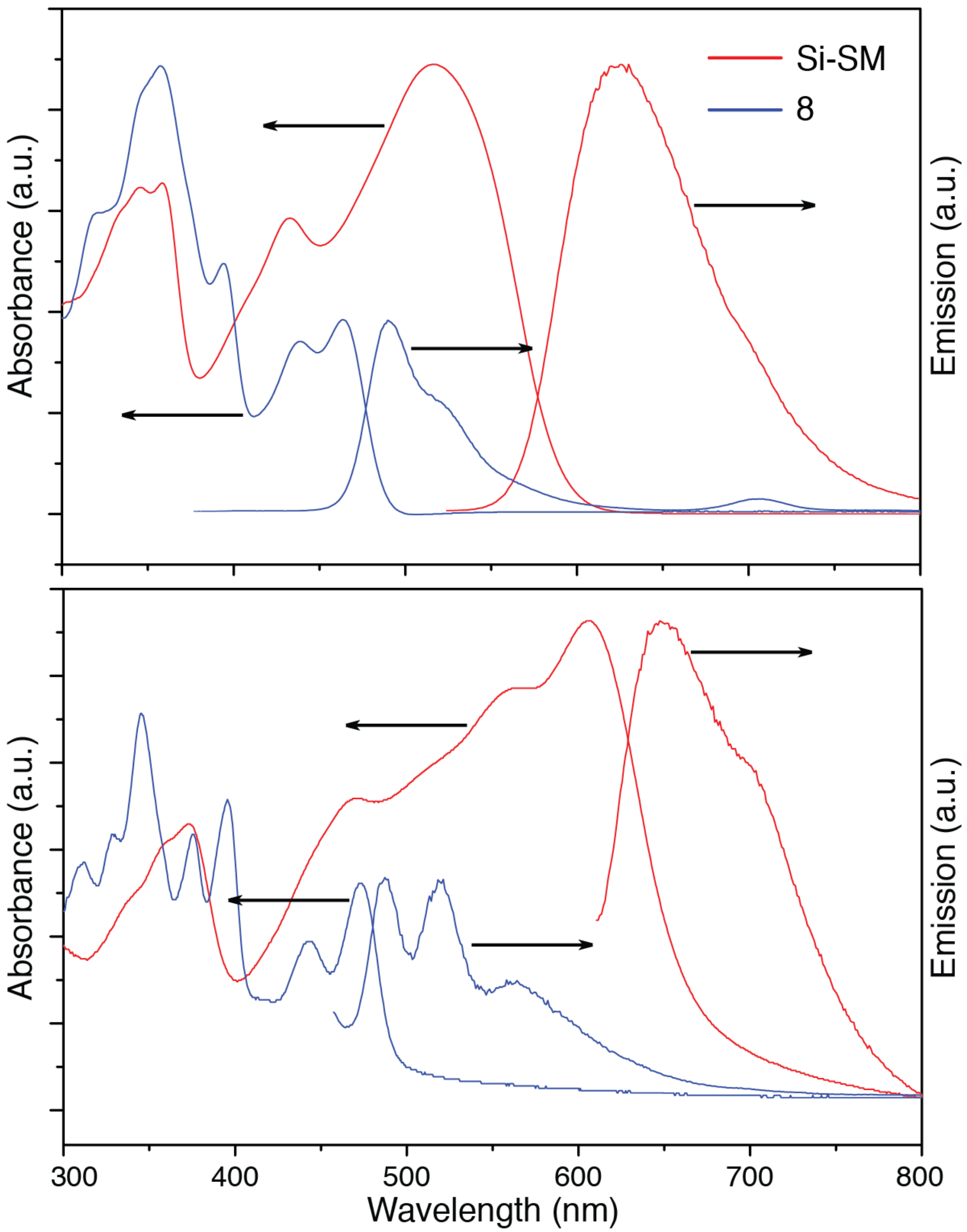

Figure S5. UV-vis absorption (left) and emission (right) spectra of compounds **Si-SM** (red) and **8** (blue): (A) in degassed chlorobenzene solutions (10^{-5} M) and (B) as thin films spun cast onto glass substrates.

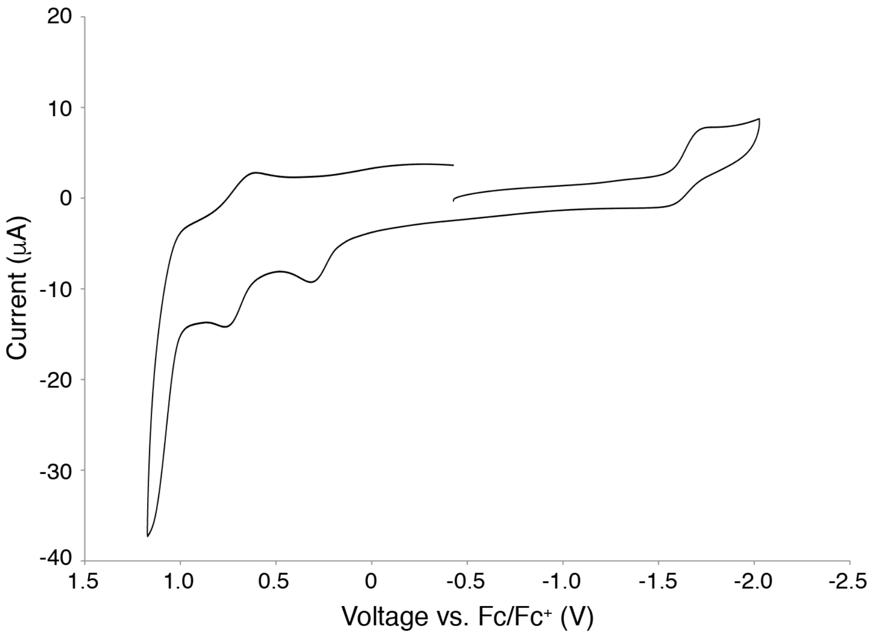

Figure S6. Cyclic voltammogram (CV) of **Pt-SM** in CH_2Cl_2 (1 mM) using Bu_4NPF_6 as supporting electrolyte (0.1 M). The potential is calibrated externally against ferrocene (Fc) redox couple (4.80 V below vacuum).

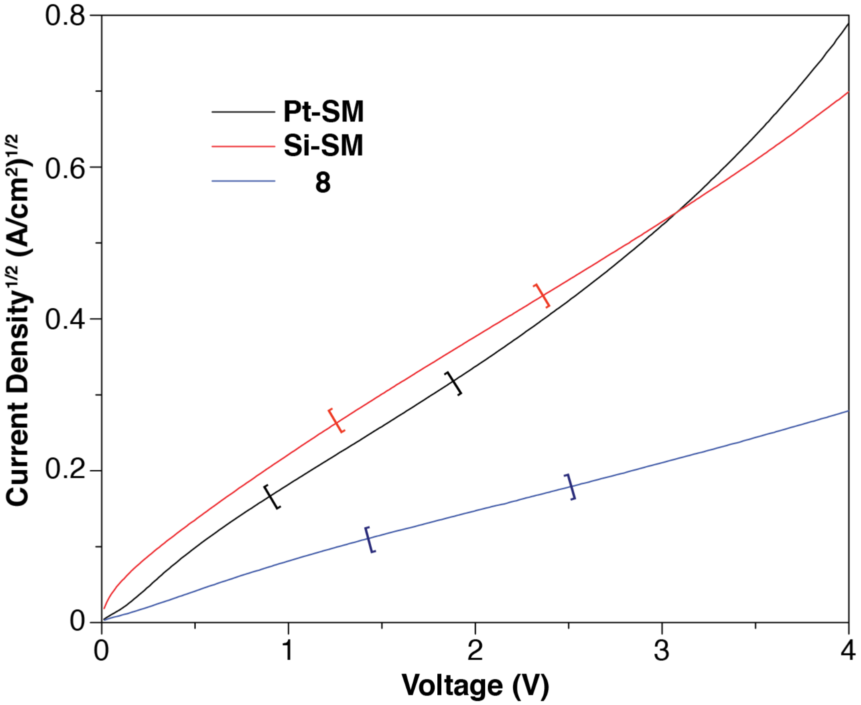

Figure S7. Space charge limited current (SCLC) profiles of **Pt-SM** (black), **Si-SM** (red) and **8** (blue) obtained from hole selective devices having geometries: ITO/MoO_3/organic/MoO_3/Al. The segments within the square brackets are the linear regions used for respective hole mobility calculations.

Table S1. Device performance parameters of **Pt-SM**/$PC_{61}BM$ devices under various fabrication conditions. Averages are calculated from at least five devices and the best values are given in parentheses.

rpm[a]	Conc.[b] mg/mL	wt./wt.[c]	Solv.[d]	Anneal.[e]	V_{OC} (V)	J_{SC} (mA/cm^2)	FF (%)	PCE (%)
1000	10	1:1	CF	AC	0.60±0.05 (0.64)	0.84±0.47 (1.55)	44.6±1.39 (46.4)	0.23±0.13 (0.43)
1000	5	1:1	CF	AC	0.84±0.02 (0.85)	3.94±0.45 (4.46)	33.3±1.56 (34.3)	1.10±0.16 (1.30)
1200	5	1:1	CF	AC	0.87±0.004 (0.87)	3.62±0.48 (4.39)	44.7±0.96 (46.2)	1.40±0.17 (1.59)
1600	5	1:1	CF	AC	0.73±0.01 (0.74)	2.68±0.37 (3.00)	53.8±2.25 (56.0)	1.09±0.14 (1.18)
1200	5	1:0.8	CF	AC	0.91±0.005 (0.92)	6.45±0.68 (7.40)	41.4±1.31 (43.2)	2.43±0.30 (2.91)
1200	5	1:0.8	CF	TA1	0.87±0.005 (0.88)	8.33±0.93 (8.90)	48.0±1.11 (49.8)	3.50[a]±0.42 (3.76)
1200	5	1:0.8	CF	TA2	0.87±0.005 (0.88)	8.33±1.50 (9.76)	52.4±4.66 (56.7)	3.80±0.74 (4.61)
1200	5	1:0.8	CF	SA1	0.87±0.005 (0.87)	8.66±1.17 (9.97)	57.8±2.29 (60.4)	4.32±0.41 (4.80)
1200	5	1:0.8	CF	SA2	0.86±0.005 (0.86)	10.66±1.51 (11.81)	56.3±0.64 (56.2)	5.14±0.70 (5.64)
1200	5	1:0.8	CF	ST	0.87±0.005 (0.87)	9.62±1.32 (10.70)	50.2±1.22 (51.8)	4.20±0.48 (4.62)

[a] Spin coating speed; all for 30s. [b] Concentration of **Pt-SM**. [c] **Pt-SM**/ $PC_{61}BM$ weight ratio. [d] Solvent; CF: chloroform. [e] Annealing conditions; AC: as cast; TA1: thermal annealing at 100 °C for 10 min after deposition of Al; TA2: thermal annealing at 120 °C for 10 min after deposition of Al; SA1: solvent (CF) annealing for 1 min before deposition of Al; SA2: solvent (CF) annealing for 2 min before deposition of Al; ST: solvent (CF) annealing for 2 min before deposition of Al and thermal annealing at 100 °C for 10 min after deposition of Al.

Table S2. Optimized performance parameters of **Si-SM**/$PC_{61}BM$ and **8**/$PC_{61}BM$ devices. Averages are calculated from at least five devices and the best values are given in parentheses. Annealing condition for both is thermal at 150 °C for 10 min after deposition of Al.

	rpm[a]	Conc.[b] mg/mL	wt./wt.[c]	Solv.[d]	V_{OC} (V)	J_{SC} (mA/cm^2)	FF (%)	PCE (%)
**Si-SM**	800	5	1:0.8	CF	0.95±0.008 (0.96)	6.37±1.16 (7.35)	52.8±3.44 (56.2)	3.20±0.44 (3.44)
**8**	800	10	1:1	CF	0.35±0.013 (0.36)	0.059±0.006 (0.058)	49.5±5.03 (55.1)	0.01 (0.01)

[a] Spin coating speed; all for 30s. [b] Concentration of **Si-SM** or **8**. [c] **Si-SM** or **8**/ $PC_{61}BM$ weight ratio. [d] Solvent; CF: chloroform.

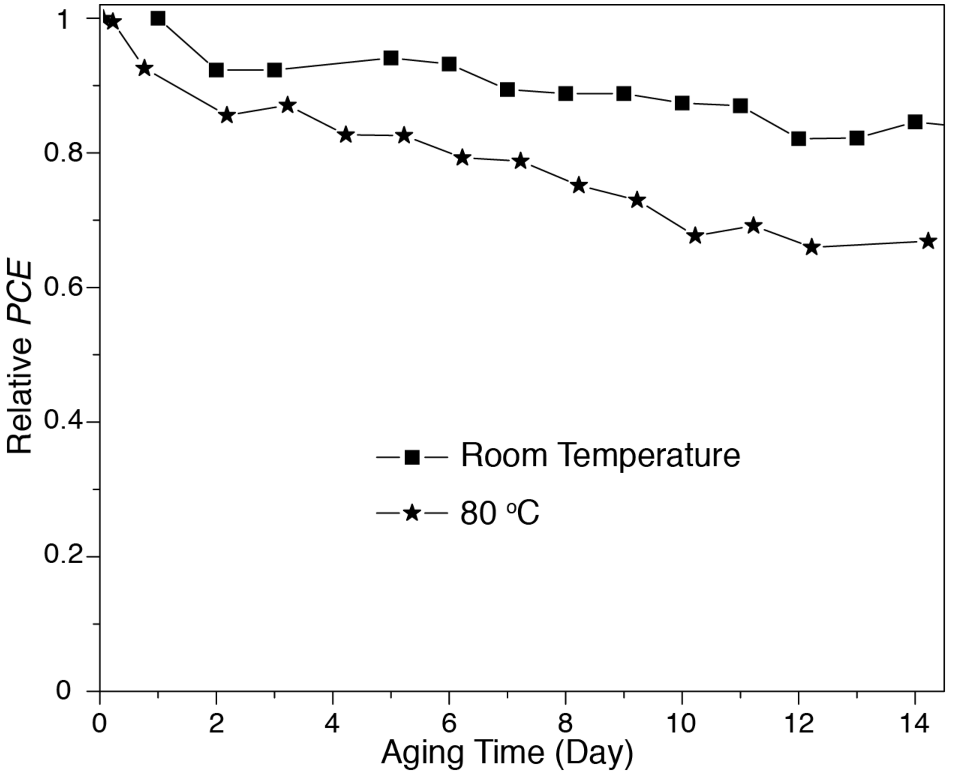

Figure S8. Aging tests of optimized **Pt-SM**/$PC_{71}BM$ devices at room temperature and at 80 °C.

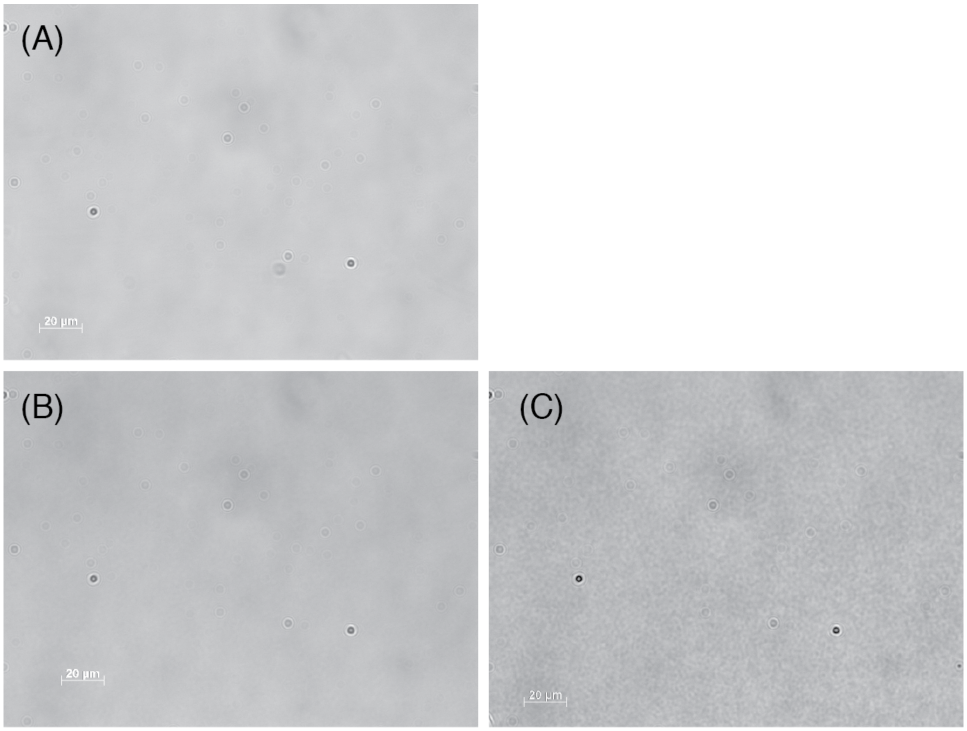

Figure S9. Optical micrographs of **Pt-SM**/$PC_{71}BM$ devices (A) as optimized; (B) aged at room temperature for 14 days; and (C) aged at 80 °C for 14 days. Scale bar: 20 μm.

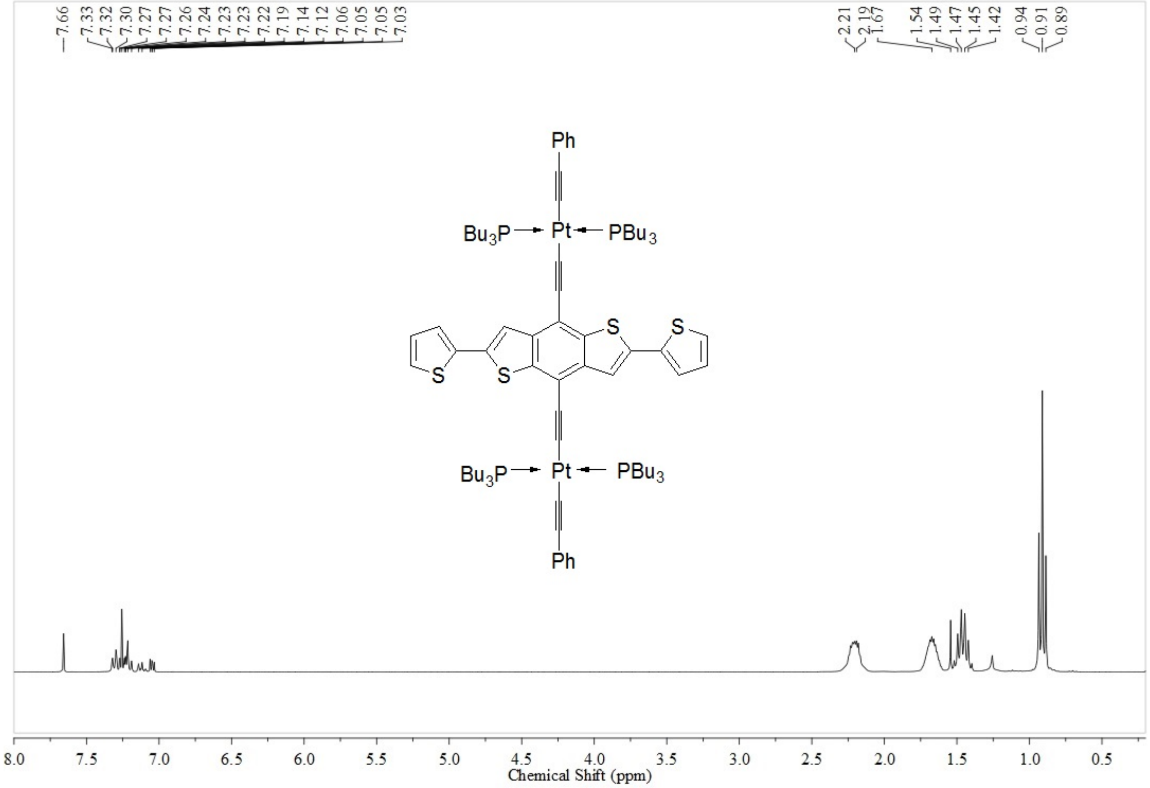

^{1}H NMR spectrum of compound **8**.

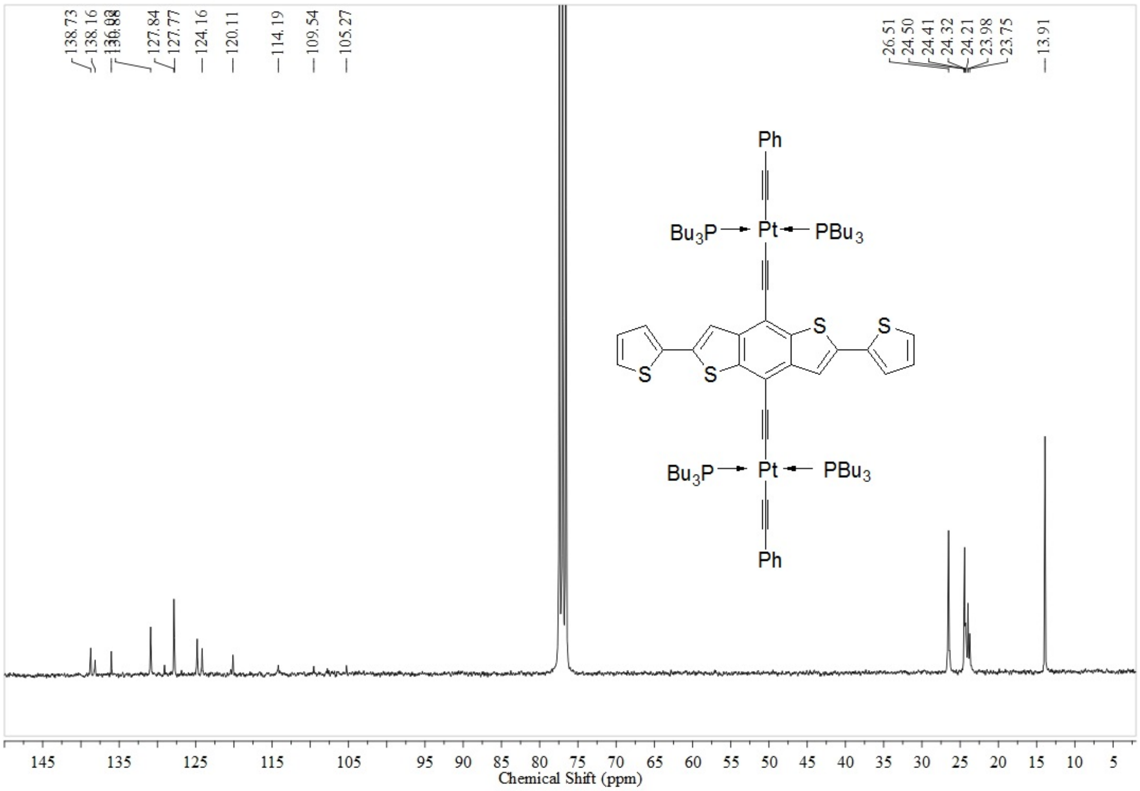

^{13}C NMR spectrum of compound **8**.

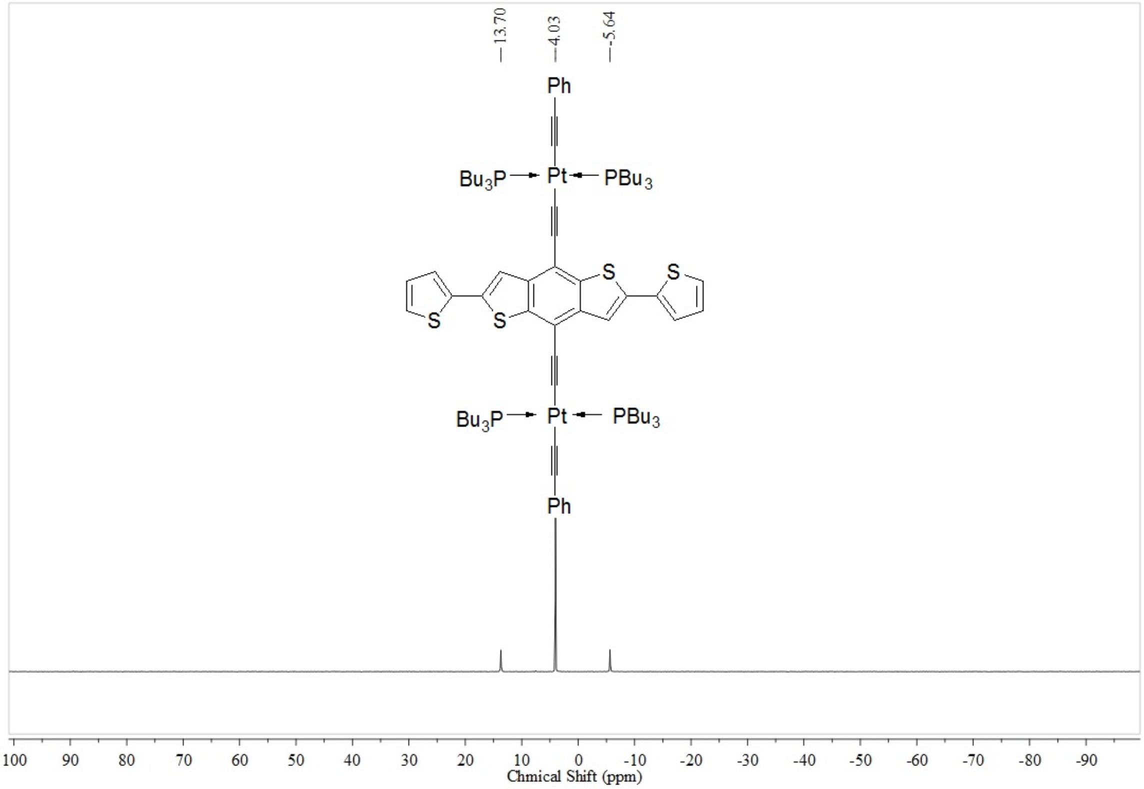

^{31}P NMR spectrum of compound **8**.

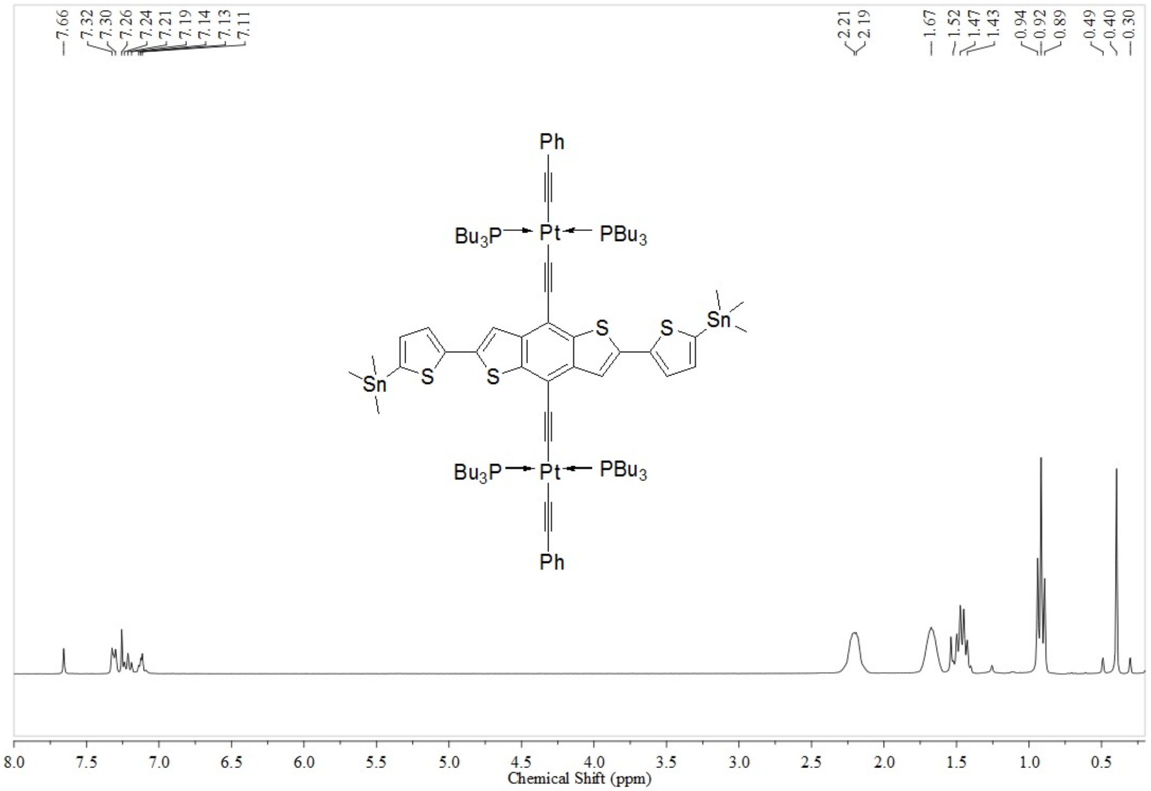

^{1}H NMR spectrum of compound 9.

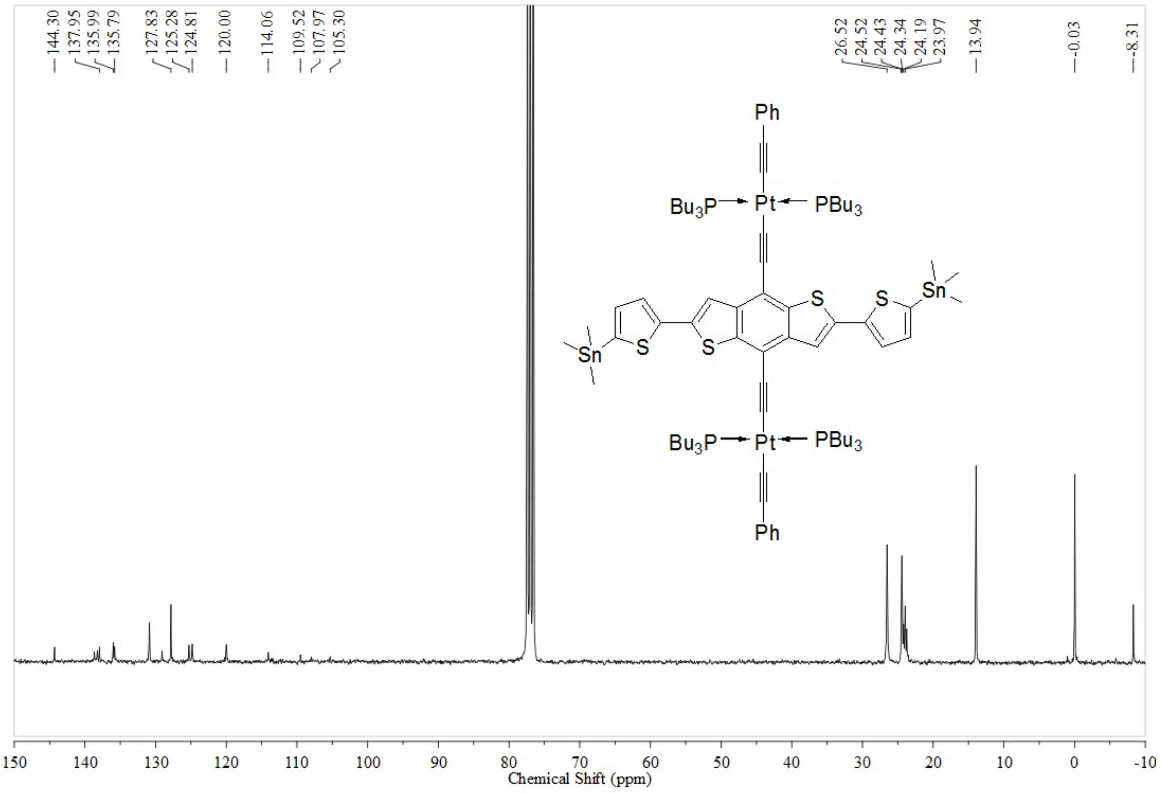

^{13}C NMR spectrum of compound 9.

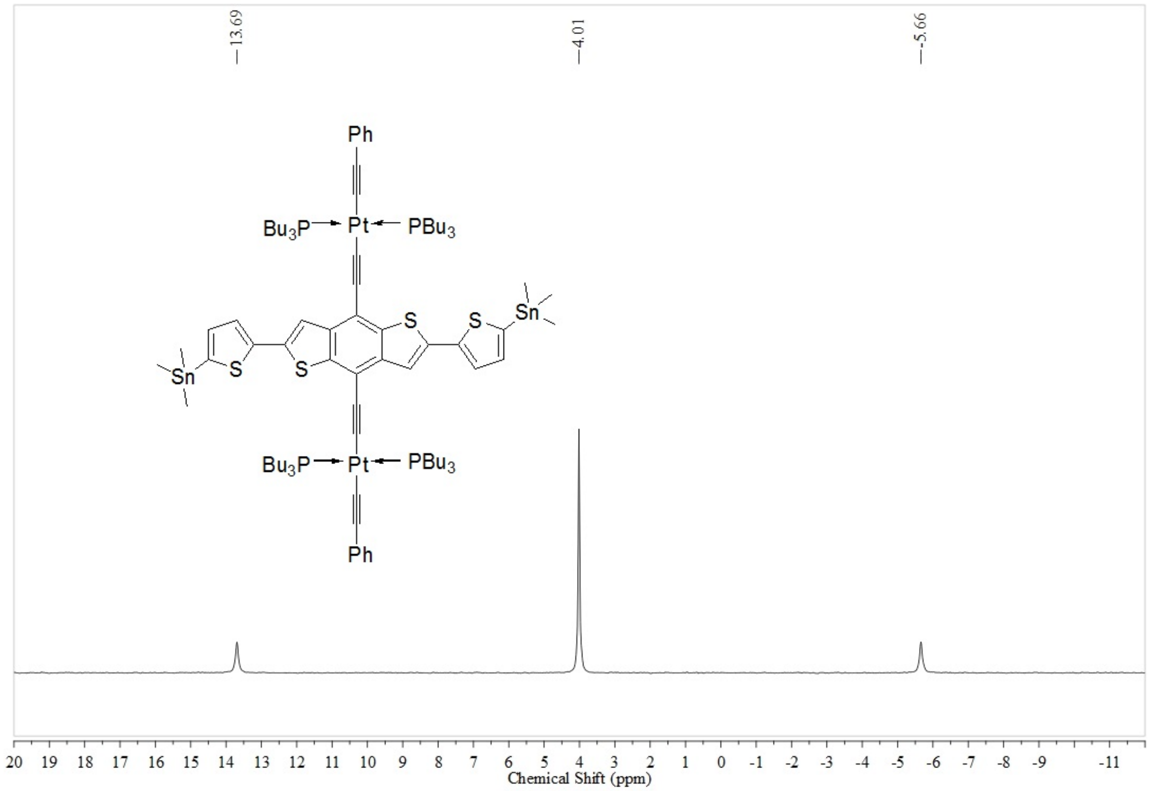

^{31}P NMR spectrum of compound 9.

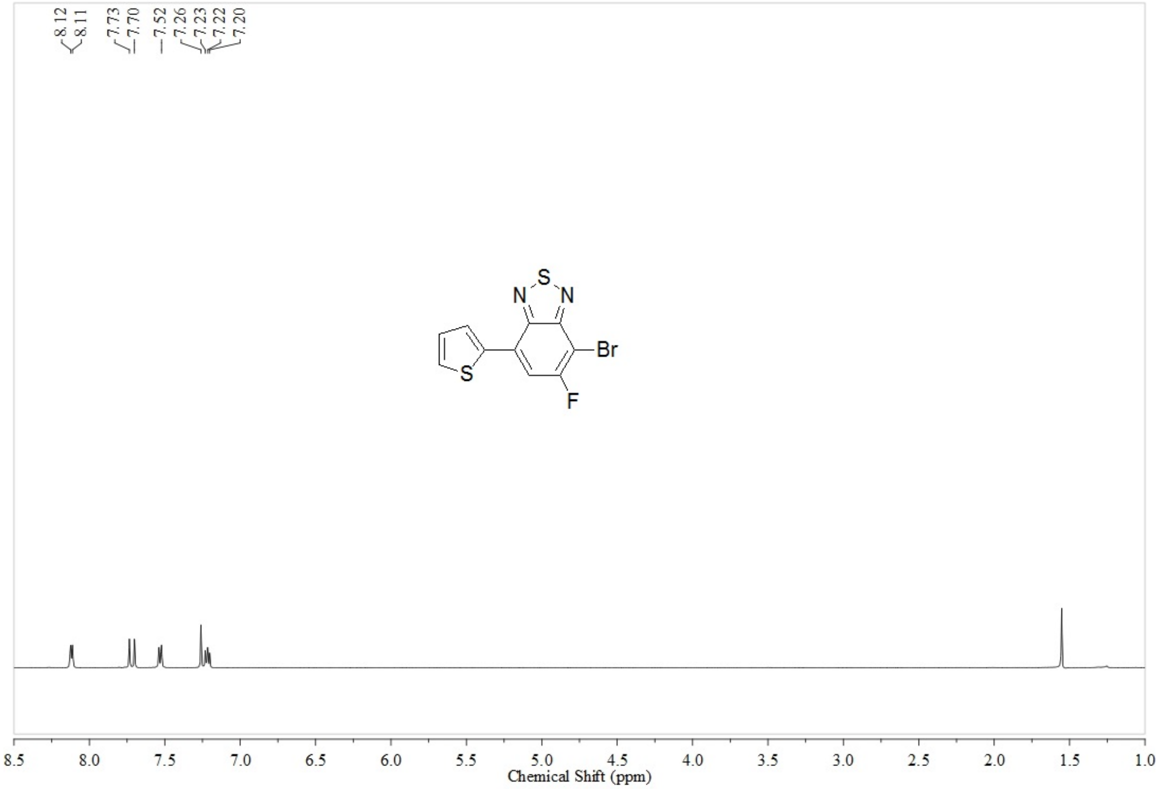

^{1}H NMR spectrum of compound 12.

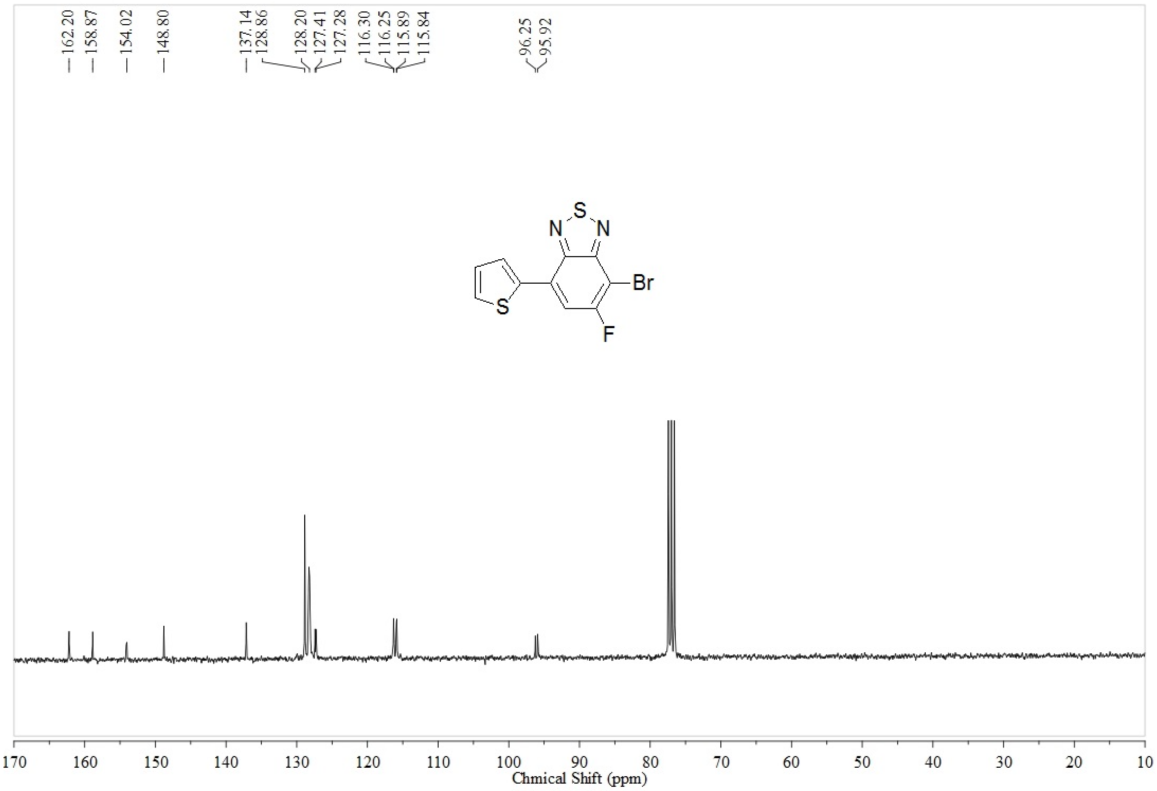

^{13}C NMR spectrum of compound 12.

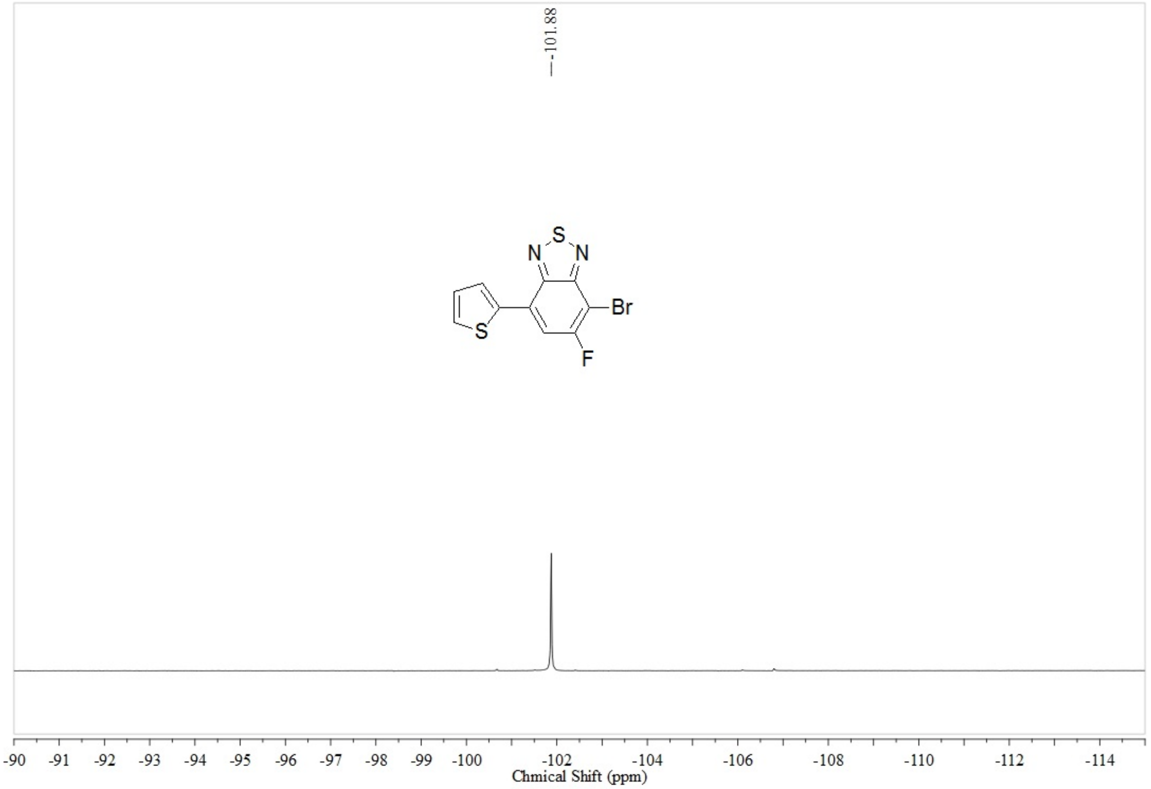

^{19}F NMR spectrum of compound 12.

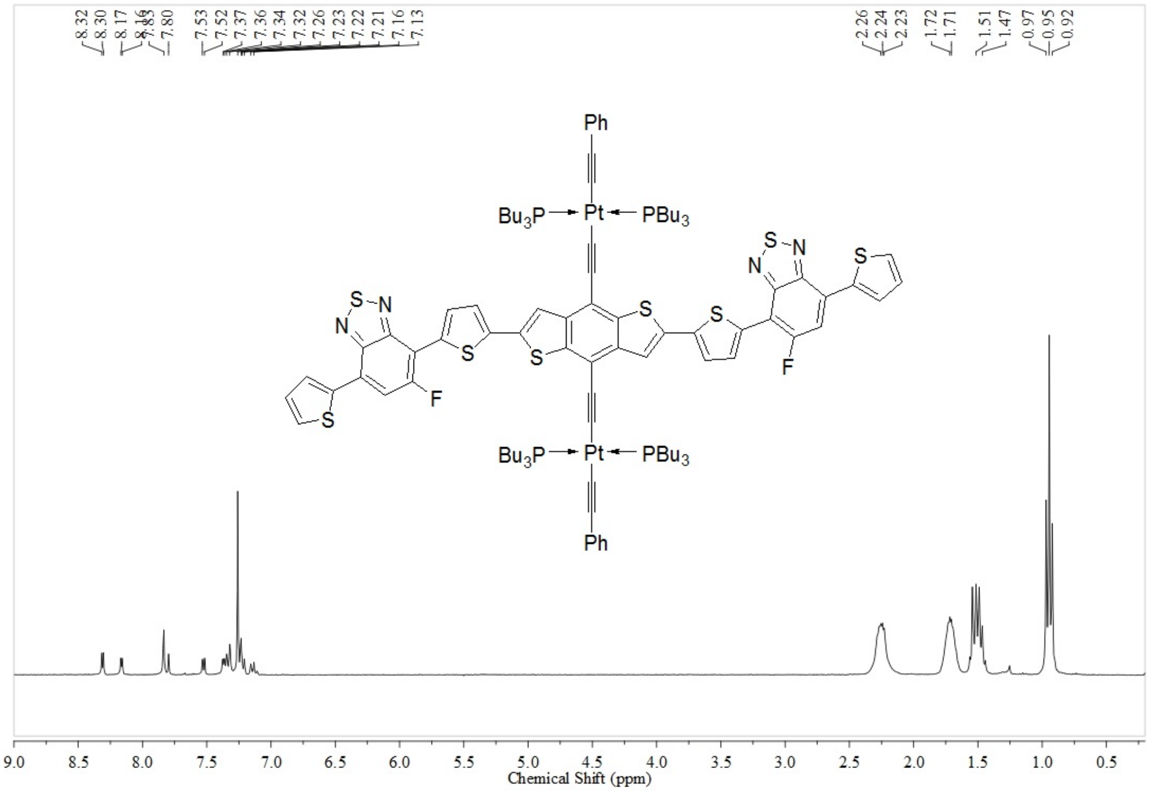

^{1}H NMR spectrum of compound **Pt-SM**.

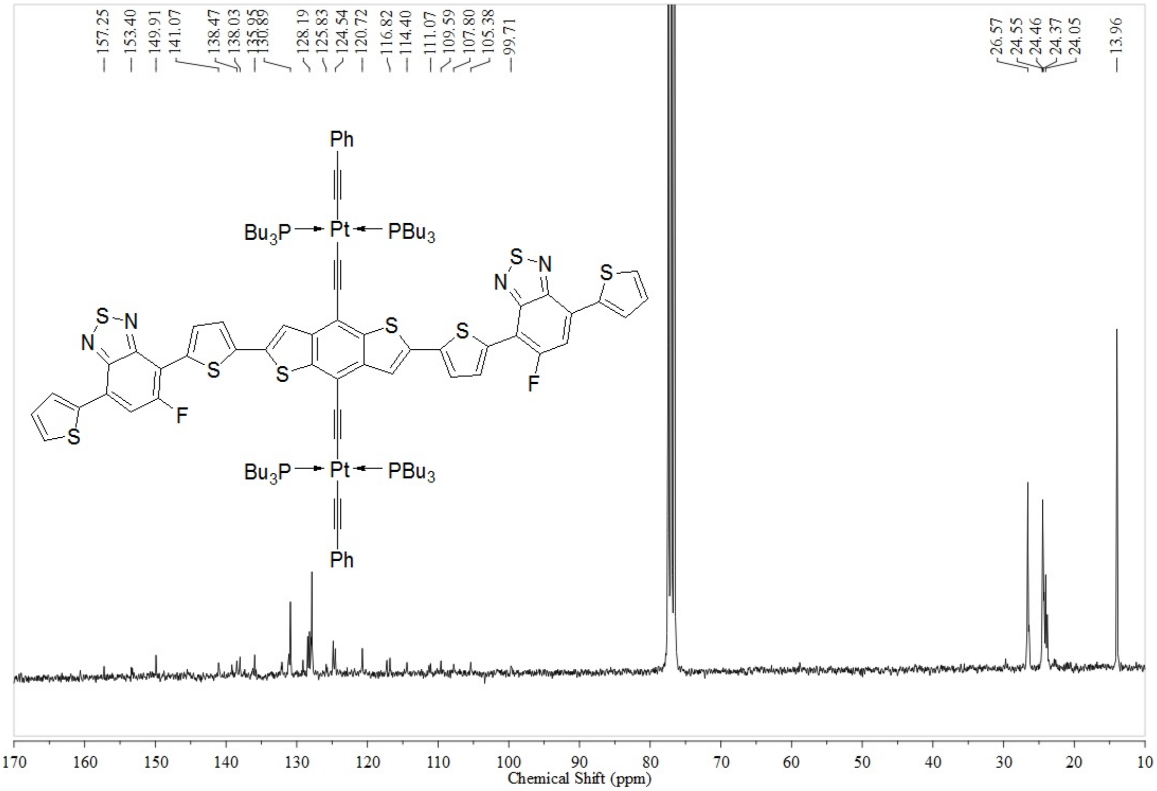

^{13}C NMR spectrum of compound **Pt-SM**.

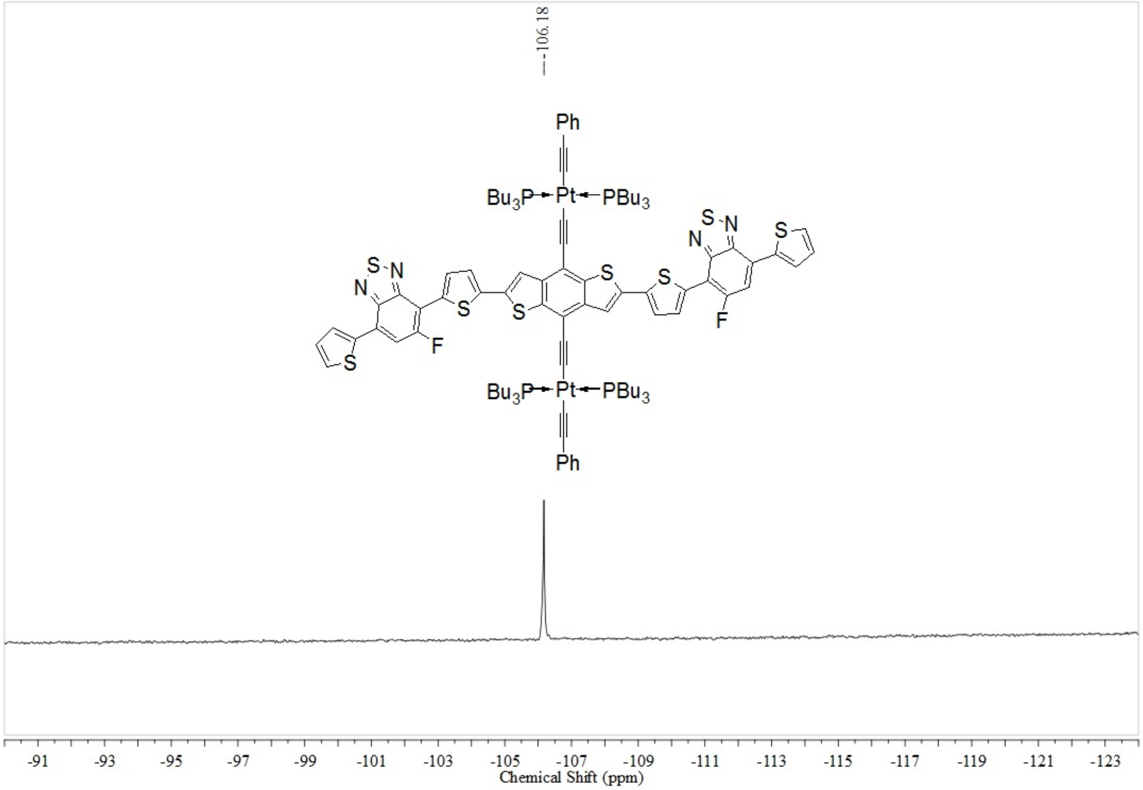

^{19}F NMR spectrum of compound **Pt-SM**.

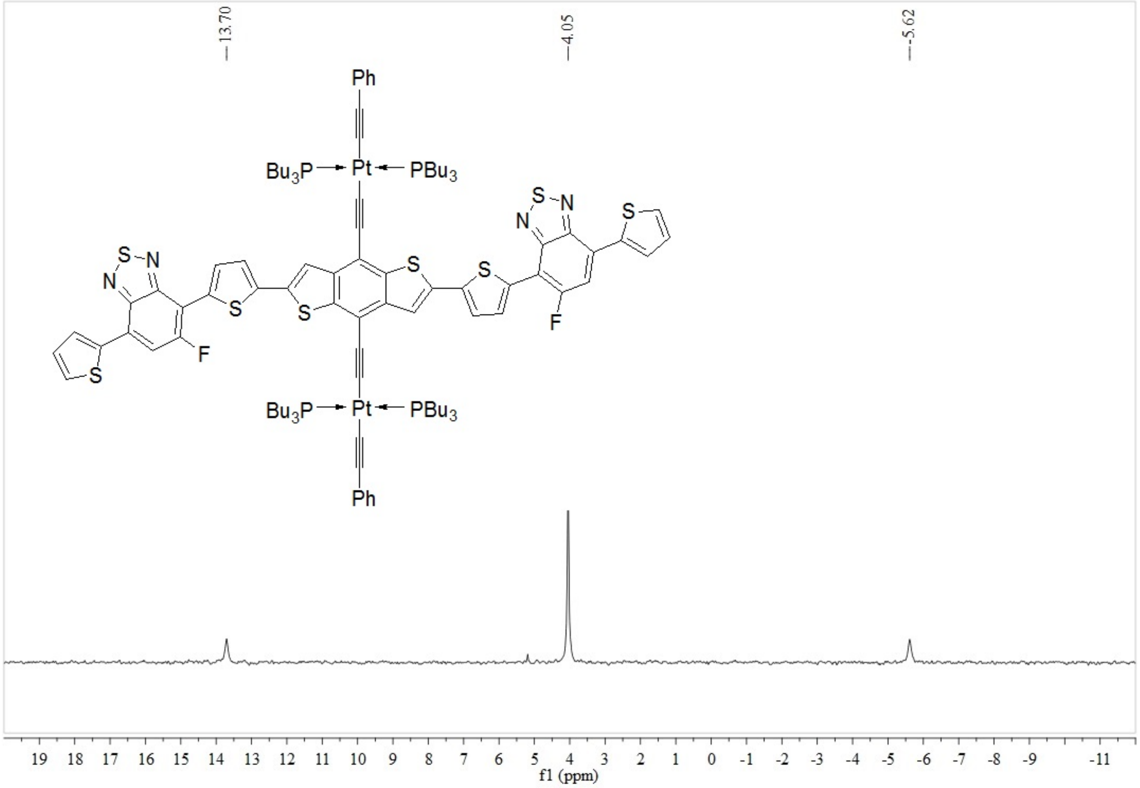

^{31}P NMR spectrum of compound **Pt-SM**.
